# Sexual and reproductive health and maternal welfare among Afghan refugee women in Pakistan: a systematic review

**DOI:** 10.3389/fgwh.2026.1645605

**Published:** 2026-07-07

**Authors:** Emily Rotondi, Rashmina J. Sayeeda, Carly Ching, David Flynn, Carrie Preston, Muhammad H. Zaman

**Affiliations:** 1 Center on Forced Displacement, Boston University, Boston, MA, United States; 2 Department of Political Science, Boston University, Boston, MA, United States; 3 Department of Biomedical Engineering, Boston University, Boston, MA, United States; 4 Department of Medical Sciences and Education, Boston University, Boston, MA, United States; 5 Department of English, Boston University, Boston, MA, United States; 6 Women, Gender, and Sexuality Studies Program, Boston University, Boston, MA, United States

**Keywords:** Afghan refugee, maternal welfare, Pakistan, sexual and reproductive health, systematic review, women

## Abstract

**Introduction:**

Women and children make up over 70% of Afghan refugees worldwide. Sexual and reproductive health (SRH) is essential for overall well-being, yet Afghan refugee women living in Pakistan face significant barriers to accessing health services. Limited access to this care contributes to numerous negative health outcomes and can have potentially lasting effects on subsequent generations. This review synthesizes evidence on the experiences, knowledge, and challenges faced by Afghan refugee women in accessing SRH services, focusing on maternal welfare.

**Materials and methods:**

Following Preferred Reporting Items for Systematic Reviews and Meta-Analyses (PRISMA) guidelines, this systematic review includes qualitative, quantitative, and mixed-methods studies from January 2000 to January 2026. Searches were conducted in databases including Boston University Online Library Database, PubMed, Science Direct, Web of Science, Embase, and EBSCOhost. Two authors independently screened titles, abstracts, and full-texts followed by completing data extraction. The quality of the study was assessed using the Mixed Methods Appraisal Tool.

**Results:**

The search yielded 679 initial search results, of which 21 studies met the inclusion criteria. Findings highlight a lack of education and awareness regarding medical and pharmaceutical contraceptives and reproductive tract infections. Afghan refugee women were found to face significant barriers to accessing healthcare, including distance, cost, stigma, and inadequate SRH education. 67% of maternal deaths were deemed preventable, and 81% of women who died from maternal-related causes faced barriers to accessing care. This population group experiences a high rate of illiteracy (93.3%), which contributes to difficulties accessing educational materials about pregnancy risk factors, leading to increased instances of pregnancy loss. Few studies examined the effects of long-term SRH interventions, and only one study analyzed how Afghan women in Pakistan exhibit resilience and self-agency in the face of structural oppression.

**Conclusion:**

This review identified key areas of limited research on SRH services and maternal welfare for commonly overlooked demographics within Afghan refugees in Pakistan, such as women of reproductive age. While maternal health outcomes of Afghan refugees have improved since 2000, women still face limited access to essential SRH services, ultimately impacting their well-being and that of future generations.

**Systematic Review Registration:**

https://www.crd.york.ac.uk/PROSPERO/view/CRD42024577910, PROSPERO CRD42024577910.

## 1 Introduction

Afghan refugees represent one of the largest and most protracted displaced populations, numbering 6.4 million worldwide, with approximately 90% hosted by Iran and Pakistan (1–3). Pakistan is home to an estimated 3.1 million refugees from Afghanistan who have sought refuge across borders due to conflict, environmental disasters, poverty, and political unrest since the late 20th century (1, 4). As a result of waves of Afghan migration into Pakistan, occurring since 1979, many Afghans reside along the Pakistani border shared with Afghanistan in the provinces of Khyber Pakhtunkhwa and Balochistan (5). Of these forcibly displaced individuals from Afghanistan, over 80% are women and children, who are often disproportionately affected by displacement as they require specialized sexual and reproductive health services (6, 7). Further, displacement with frequent periods of movement has been attributed to the lack of healthcare infrastructure and access to necessary sexual and reproductive healthcare for refugees (8).

Due to the difficulty accessing these services, refugee and displaced women and children are placed at a higher risk for adverse health outcomes, such as unintended pregnancy, complications during pregnancy and childbirth, and a greater risk of contracting sexually transmitted infections (9, 10). Nevertheless, Afghan women have demonstrated active resistance to the challenges they face through involvement in community leadership, participation in economic activity, and creation of community networks. Contrary to notions, Afghan women are active agents within their communities and exert self-agency to navigate structural oppression and ensure the survival of themselves and their community (11).

Thus, research on the accessibility, experiences, and challenges of obtaining sexual and reproductive healthcare for Afghan refugees is vital for enhancing future studies and identifying areas for improving the existing public health systems. However, despite the size of the Afghan refugee population residing in Pakistan, few systematic reviews have examined the SRH and maternal welfare of Afghan refugees in Pakistan. The existing reviews that have examined the health of Afghan refugees focused on Afghan and Rohingya refugees across Asia (12), Afghan refugees living in Iran (13, 14), the mental health of Afghan refugees resettled in Western countries (15–18), and the health of Afghan refugees living in Germany (19). To our knowledge, no existing review has analyzed both the maternal welfare and SRH of Afghan refugees in Pakistan, considering topics such as mental health, nutrition, and abortion access. This review aims to synthesize the available evidence on research trends, gaps, understudied topics, and patterns in the existing research on the maternal welfare and SRH of Afghan refugees in Pakistan.

## 2 Methods

### 2.1 Review focus and guidelines

This review was registered in the PROSPERO database (ID CRD42024577910). This systematic review examined peer-reviewed literature on maternal welfare as well as sexual and reproductive health experiences of Afghan refugee women in Pakistan, following the PRISMA guidelines.

### 2.2 Search strategy and selection criteria

This review included relevant articles from the following databases: Boston University Online Library Database, PubMed, Science Direct, Web of Science, Embase, and EBSCOhost. The complete search strategy is available in the Supplementary Appendix. Literature searches were performed three times to identify and capture relevant literature: first in February 2024, then on August 18, 2024, and finally updated on January 22, 2026. Furthermore, the reference lists of included articles were examined for relevant studies. Grey literature sources were not included in this review. Qualitative, quantitative, and mixed-method studies were included in the review to ensure a comprehensive analysis of existing literature. The inclusion and exclusion criteria are outlined in Table 1.

**Table 1:** Inclusion and exclusion criteria.

Category	Included	Excluded
Population of interest	Afghan refugee women of reproductive age, mothers, and newborns in Pakistan	Non-Afghan refugee populations or studies conducted outside of Pakistan
Publication date	Studies published from 2000 to January 2026	Studies published before 2000 or after January 2026
Study design	Qualitative, quantitative, and mixed methods studies	Letters, commentaries, editorials, grey literature, and reviews (though references were screened for additional articles)
Language	Articles published in English	Studies in non-English languages
Topics	Papers describing sexual and reproductive health (SRH), including maternal health, newborn and child health, abortion care, STIs, nutrition, mental health, and gender-based violence	Studies unrelated to SRH and or maternal welfare.

The search was conducted using specific terms related to maternal welfare and reproductive health, covering literature published from January 2000 to January 2026. Senior Research Librarian (DF) was consulted on developing the protocol and search strategy.

The search terms used are detailed in Table 2. Our search strategy used the following format: [(population search term) AND (status search terms) AND (demographic search terms) AND (topic search terms) AND Pakistan]. The choice of search terms and topics used was guided by the SRH definitions from the International Conference on Population and Development in 1994 (20).

**Table 2:** Searched terms used in online databases.

Category	Search terms
Population	Afghan
Status	Afghan migrant OR Afghan refugee OR Afghan refugee women OR asylum OR displaced OR displaced person OR migrant OR refugee OR refugee women
Demographic	Adolescence OR adolescent OR female OR girl OR teen OR teenager OR woman OR women OR young female OR young marriage OR young person OR young women OR youth
Topic	access to maternal healthcare OR AIDS OR antenatal OR antenatal care OR birth OR birth complications OR birth outcomes OR child marriage OR condom OR contraception OR contraceptive use OR dowry death OR early marriage OR early motherhood OR family health OR family planning OR forced marriage OR forced sex OR health outcomes OR HIV OR maternal OR maternal health OR maternal healthcare access OR maternal mortality OR maternal nutrition OR maternal well-being OR menstrual hygiene OR menstruation OR mental health OR mortality rate OR motherhood OR neonatal mortality OR nutrition OR obstetric care OR perinatal outcomes OR physical relationship OR post-natal OR post-natal care OR pregnancy OR pregnancy complications OR rape OR relationship OR reproductive OR reproductive health OR reproductive health care OR reproductive health issues OR reproductive morbidity OR reproductive rights OR safe childbirth OR sexual activity OR sex OR sex education OR sexual OR sexual abuses OR sexual assault OR sexual behavior OR sexual coercion OR sexual experience OR sexual health OR sexual initiation OR sexual intercourse OR skilled birth attendants OR suicide OR teenage pregnancy OR violence OR woman's health
Location	Pakistan

### 2.3 Screening and data extraction

**Figure 1:**
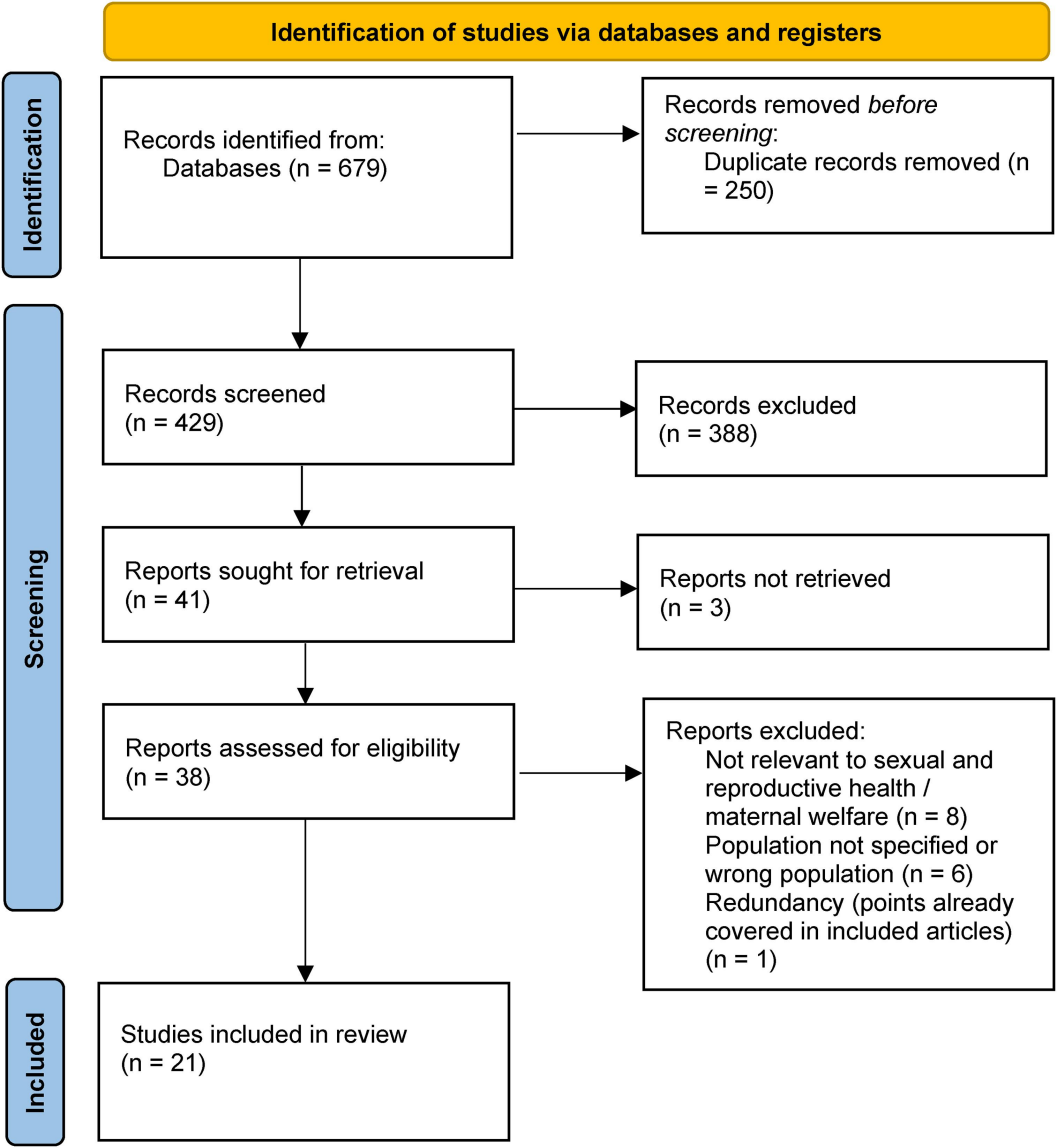
PRISMA flowchart.

All articles were imported into Rayyan, an artificial intelligence tool for systematic reviews (21). After removing duplicates on Rayyan, titles and abstracts were manually screened for inclusion. Two authors (ER and RJS) blindly reviewed and screened the articles for quality and relevance. Discrepancies were resolved via discussion between the two authors. To avoid reporting bias, both authors extracted information independently. 679 articles were initially screened, and 429 articles remained after deduplication. Following title and abstract screening, 38 articles were selected for full-text review, of which 21 met the inclusion criteria. The PRISMA flowchart (Figure 1) summarizes the search results. Supplementary Table S4 in the appendix details the studies that were excluded during full-text screening, with reasons for exclusion.

Upon full review of the included articles, data were extracted on regional focus, methodology, study participants, location of authors, funding source, authors' disciplines, study purpose, key results, ethical considerations, community involvement, and study limitations into a standardized spreadsheet. The data and findings were then categorized by common themes, resulting in a thematic summary of the 21 articles.

### 2.4 Quality assessment

The Mixed Methods Appraisal Tool (MMAT) was used to assess the quality and risk of bias of the included studies. This tool assesses the research designs of qualitative, quantitative, and mixed-methods studies (22). The MMAT asks two initial screening questions: whether there are clear research questions and whether the data collected allow those questions to be answered. If these questions are not satisfied, the study may not be eligible for appraisal. The MMAT scoring ranges from low quality (0/5 and 1/5) to moderate quality (2/5 and 3/5) to high quality (4/5 and 5/5). The scoring for each study is based on the evaluation of study selection bias, study design, data collection methods, sample size, intervention integrity, and analysis. Two authors (ER and RJS) independently completed the data quality analysis. Discrepancies between authors were discussed until a consensus was reached.

## 3 Results

### 3.1 Article characteristics

Twelve out of 21 articles (57%) (23–34), focused on Afghan refugees living in Khyber Pakhtunkhwa (formerly known as the North-West Frontier Province), covering areas such as Hangu, Jalozai Camp, Kohat, Peshawar, Haripur, New Shamshatoo, Shalman, Dera Ismail Khan, Bannu, and Azakhel refugee camp in Noshwera. Three articles (14%) were conducted in Balochistan, specifically in Quetta City (35–37). Two articles (10%) examined Afghan refugees across both Khyber Pakhtunkhwa and Balochistan (11, 38). The remaining articles focused on Afghan refugees in other areas of Pakistan, including Karachi (39), Islamabad (40), Karachi and Quetta (41), and Islamabad, Quetta, Karachi, and Peshawar (42) (Table 3). Of the studies included in this review, 12 (57%) were quantitative (23, 24, 30–32, 34, 37–42), five (24%) were qualitative (11, 25, 29, 33, 35), and four (19%) were mixed-method studies (26–28, 36).

**Table 3:** Details of studies included in this review.

Study title	Citation	Regional focus and setting	Data theme	Study design	Author(s) location	Key findings	Sample size (*N*)	Key predictor	OR	Population	Author identified critical limitations
Risk factors for low birth weight in the public hospitals at Peshawar, NWFP-Pakistan	Badshah et al. (2008) (34)	Peshawar, Khyber Pakhtunkhwa	Abortion	Cross-sectional prospective survey	Pakistan, United Kingdom	The main geo-demographic risk factors for small for gestational age (SGA) identified in this study, controlling for gestational age of less than 37 weeks, are maternal age, nationality, and consanguinity. Presentation with anemia and the history of previous abortion/miscarriage were also found to be significant independent factors. The adjusted odds ratio for gestational age showed the largest effect in explaining the incidence of low birthweight LBW. The next highest odds ratio was for maternal age below 20 years. The explanatory model included two pairwise interactions, for which the predicted incidence figures for LBW show an increase among the Tribal area with presentation of anemia, and among full-term babies with their mothers having a previous history of abortion/miscarriage.	1,039	Gestational age	6.4	Single birth mothers from 4 public birth hospitals in Peshawar	Because the study was unable to interview women in privacy, they could not determine induced vs. spontaneous abortions. Due to the limited participation of clinicians, the study could not collect information from all mothers admitted for delivery in the four hospitals from August to November. The findings of this study are specific to public hospitals in Khyber Pakhtunkhwa, Pakistan.
Human security and sustainable development goals: the voices of Afghan women refugees in Pakistan	Bakare et al. (2025) (33)	Kohat, Khyber Pakhtunkhwa	Gender-based violence,	Qualitative study	Pakistan	The key findings suggest that women in camps have cocooned lives, and their patriarchal cultural set-up perpetuates gender-spatial segregation, which consequently limits women's opportunities to access and traverse spaces other than their immediate residential location (camps). In addition, they are deprived of seeking education outside the radius of the camps and are not allowed to have mobile phones.	40	N/A	N/A	Afghan refugee women in refugee camps in Kohat	Not reported
Reproductive tract disorders among Afghan refugee women attending health clinics in Haripur, Pakistan	Balsara et al. (2010) (30)	Haripur,Khyber Pakhtunkhwa	Reproductive tract infections	Qualitative study	United States of America, Pakistan	Over three-fourths (76.7%) of those who reported to the health clinics with reproductive complaints had an RTI. Nearly half (49.5%) of these women were diagnosed with some form of vaginitis, and 14.7% were diagnosed with clinical suspicion of pelvic inflammatory disease (PID). Women with cervical prolapse (*p* = 0.033) or who cleansed after intercourse (*p* = 0.002) were more likely to have vaginitis. There was a significant difference (*p* = 0.017) in the prevalence of suspected PID among women who used mud only (11.1%), any water (18.8%), and an old cloth or toilet paper (9.8%) for cleansing after defecation.	634			Afghan refugee women, aged 12–70 years, living in refugee villages in Haripur, Pakistan, who attended a Basic Health Unit for reproductive health-related complaints.	Selection bias in the enrollment process, as those interviewed were women who self-reported to Basic Health Units for reproductive health complaints. Thus, the results of this study cannot be extrapolated to the Afghan refugee community as a whole. The study also excluded those women who had been on antibiotics. These women may have had more financial resources to seek care.
Maternal mortality among Afghan refugees in Pakistan, 1999–2000	Bartlett et al. (2002) (23)	Near Hangu in Khyber Pakhtunkhwa	Gender-based violence, maternal and neonatal mortality	Population-based retrospective cohort study	United States of America, Pakistan	The census identified 134,406 Afghan refugees and 1,197 deaths: a crude mortality rate of 5·5 (95% CI 5·2–5·8) per thousand population. Among the 66 deaths among women of reproductive age, deaths due to maternal causes (*n* = 27) exceeded any other cause (41% [95% CI 29–53]). 16 liveborn and nine stillborn infants were born to women who died of maternal causes; six of the liveborn infants died after birth. Therefore, 60% (15 of 24) of infants born to these women were either born dead or died after birth. Compared with women who died of non-maternal causes, women who died of maternal causes had a greater number of barriers to health care (*p* = 0.001), and their deaths were more likely to be preventable (*p* < 0.05).	134,406			12 Afghan refugee settlements near the village of Hangu, in Khyber Pakhtunkhwa, Pakistan	Not all deaths may have been identified in the census, resulting in lower study mortality estimates. Age and sex-specific mortality rates were not available, making it difficult to assess the overall mortality situation. Misclassification of cause of death during verbal autopsy interviews is possible, since medical records were not available for all women, nor were researchers able to interview health care providers.
Nutritional and health status of Afghan refugee women living in Punjab: A cross-sectional study	Fatima et al. (2023) (40)	Islamabad	Nutrition, reproductive tract infections	Cross-sectional study	Pakistan, Iraq	The results indicate the prevalence of underweight, normal weight, and overweight at 74.7%, 16.7%, and 8.7%, respectively. The majority of the women have extremely low hemoglobin (Hb) levels, which indicates iron deficiency as well as low body mass index for their age. The results indicate that there are high chances of severe malnutrition.	150			Afghan refugee women aged 15–30 years, living in sector H-12 of the city of Islamabad, where about 2,000–3,000 Afghan refugees live in muddy houses	Not reported
Suicidal feelings run high among mothers in refugee camps: a cross-sectional survey	Rahman and Hafeez (2003) (31)	Khyber Pakhtunkhwa Shamshatu and Shalman refugee camps	Mental health	Cross-sectional survey	Pakistan	One hundred and six (36%) of women in the sample screened positive for a common mental disorder. Ninety-six (91%) of those screening positive had had suicidal thoughts in the previous month, and nine (8%) rated suicidal feelings as their topmost concern.	297			Afghan refugee mothers with children located in Shamshatu and Shalman refugee camps	Not reported
Integrating health care for mothers and children in refugee camps and at district level	Hafeez et al. (2004) (26)	New Jalozai, New Shamshatoo, and Shalman camps Case Study 2: Nowshera and Gujranwala districts	Maternal and neonatal mortality	Qualitative case-study	Pakistan, United Kingdom	Health care for mothers and children is inadequate in most refugee situations and in poorly resourced countries. The authors argue that, as well as providing primary (home-based) care for basic health care, there is a need to integrate primary care with adequately functioning hospital-based care for a healthcare system to succeed	Not reported			Afghan refugees located in the refugee camps: New Jalozai, New Shamshatu, and Shalman	Not reported
Intimate partner violence among Afghan women living in refugee camps in Pakistan	Hyder et al. (2007) (25)	Khyber Pakhtunkhwa (Jalozai Camp)	Gender-based violence	Exploratory qualitative study	United States of America	From the interviews, it is evident that women do experience violence during day-to-day conflict, and that conflict occurs not only between women and their husbands but also between women and other family members. It is also clear that health workers have little training or support to deal with cases of violence, and that further exploration of the issues surrounding day-to-day conflict is necessary to develop culturally appropriate interventions.	20 women of reproductive age and 20 health workers			Afghan refugees and health workers located in the refugee camp, Jalozai, which is located outside the province’s capital city, Peshawar	The study relied on the assistance of health workers for the recruitment of women for the study, and was not able to control for the biases of the health workers during recruitment.
Gender dynamics and the role of women in refugee communities in Pakistan: a case study of Afghan refugee camps	Ismail et al. (2025) (11)	Khyber Pakhtunkhwa and Balochistan	Gender-based violence	Qualitative study	China	The study finds that Afghan women exhibit remarkable agency, participating in informal economic activities, assuming leadership roles within their communities, and advocating for empowerment. The paper identifies key themes of survival strategies, informal support networks, and gendered power dynamics in refugee camps.	10			Afghan refugee women, ministers from local authorities, and NGO staff members	The current study area of Khyber Pakhtunkhwa, along with Balochistan, fails to offer a comprehensive understanding of the wide-ranging experiences of Afghan refugee women across all Pakistani regions and international host destinations. The understanding of Afghan women's experiences in cities and refugee settlements is limited.
The reproductive health issues & practices among the Afghan refugee women (with special reference to Afghan Basti in Karachi and Quetta)	Khan et al. (2022) (41)	Karachi and Quetta	Family planning and contraception	Qualitative study	Pakistan	It was found that many of the women who were experiencing difficulties with their reproductive health also had other health issues, such as diarrhea, gastritis, anemia, respiratory infections, “weakness,” renal problems, and skin illnesses. These issues persisted because of poor sanitation and the absence of a functional sewage system in the neighborhood. The Afghan women refugees claim to have mental health problems as well, but they claim they have not been able to access therapists or psychiatrists.	120			Afghan refugee women, aged 15–49 years, living in informal settlements in and around the cities of Quetta and Karachi	Not reported
Gender, culture, and migration: a qualitative study of the socioeconomic challenges facing Afghan women refugees in Khyber Pakhtunkhwa, Pakistan	Khan et al. (2024) (29)	Azakhel refugee camp in Noshwera, Khyber Pakhtunkhwa	Gender-based violence, maternal and neonatal mortality	Qualitative study	China, Pakistan	The research highlights challenges from forced migration, gender inequality, cultural norms, and socioeconomic marginalization, causing a holistic crisis for Afghan refugee women in Khyber Pakhtunkhwa, Pakistan. These issues hinder access to education, employment, financial vulnerabilities, and legal uncertainties.	3			Afghan women citizens, aged 22–35 years, in Azakhel refugee camp in Noshwera, Khyber Pakhtunkhwa, Pakistan.	Not reported
Comparison of a positive deviant inquiry with a case-control study to identify factors associated with nutritional status among Afghan refugee children in Pakistan	Lapping et al. (2002) (28)	Haripur,Khyber Pakhtunkhwa	Nutrition	Qualitative study	United States of America, Pakistan	The positive deviance inquiry (PDI), identified 12 feeding, caring, and health-seeking behaviors that were not widely practiced. The case-control study (CCS), yielded six significant associations with good nutritional status. Both the PDI and CCS detected feeding behaviors. The PDI alone identified complex phenomena (active feeding and maternal affect). The CCS alone confirmed the beneficial use of health services.	8 families, 50 children			Pashtun Afghan refugees living in and around the Haripur refugee camps, which are permanent settlements, two hours northwest of Islamabad	Key limitations include incomplete community verification of the Positive Deviance Inquiry conclusions, potential biomedical biases by those conducting the PDI, inconsistencies amongst the field workers, limited generalizability of specific findings since data was gathered during the summer, and variations among the ages of positive deviant and non-positive deviant children.
Disease status of Afghan refugees and migrants in Pakistan	Malik et al. (2019) (32)	Khyber Pakhtunkhwa districts of Peshawar, Dera Ismail Khan, and Bannu	Reproductive tract infections	Health status evaluation	United States of America, Pakistan	The most prevalent reported infections were respiratory tract infections (48.05%). Skin diseases and Diarrhea collectively affected 21.08% of Afghan refugees. Overall, the disease burden was higher in females than in males in the Afghan refugee population.	Not reported			Not reported	Not reported
Identification of model newborn care practices through a positive deviance inquiry to guide behavior-change interventions in Haripur, Pakistan	Marsh et al. (2002) (27)	Haripur,Khyber Pakhtunkhwa	Maternal and neonatal mortality	Qualitative study	United Kingdom, United States of America, Pakistan	The Afghan caregivers showed better use of services and some household practices than their Pakistani counterparts, consistent with the duration of Save the Children Federation/US presence (15 years vs. 18 months, respectively). The practices of both groups for clean delivery, thermal control, immediate and exclusive breastfeeding, and fathers’ involvement were weak. But PD individuals, families, and/or birth attendants modeled good maternal care and immediate, routine, and special newborn care. Communities enthusiastically committed to changing behavior and forming neighborhood support groups for better newborn care, including a demand for hygienic delivery.	Not reported			Afghan refugees located in Camp Five of Basic Health Unit 4, 10 km west of Haripur City	Not reported
Midwives providing maternal health services to poor women in the private sector: is it a financially feasible model?	Mumtaz (2021) (36)	Balochistan (Quetta City)	Maternal and neonatal mortality	Qualitative study	Canada	The single midwife-practices saw a mean of 8.7 ANC patients (range 1–19), attended 2.9 births (range 0–10), and provided care to 1.6 postnatal patients (range 0–7). The average net income of the 11 practices in May 2014 was US$81, but the median was just US$12. To contextualize these incomes, the midwives earned, on average, 25% of Pakistan’s minimum monthly living wage. The financial analysis showed that only 3 out of 11 sampled practices could be considered financially viable. The qualitative data revealed that even in practices with reasonable client volumes, the patient's inability to pay was the critical factor in the midwife practices’ low net incomes. The research provides empirical evidence of a potential pitfall of private funding models in resource-poor settings where providers rely on impoverished clients to pay user fees.	11			Afghan refugee women trained as midwives in Quetta City, Baluchistan, who were recruited primarily from informal settlements around the city.	Due to an uncertain security situation in the refugee camps in Quetta city and the province of Baluchistan generally, the researchers were unable to have a prolonged presence in the field site. The program also lost contact with many of its midwife alumni, largely because Afghan refugees are a very mobile population. These limitations created limits on follow-up interviews and sample size.
Reducing maternal mortality among Afghan refugees in Pakistan	Purdin et al. (2009) (24)	Hangu (Khyber Pakhtunkhwa)	Maternal and neonatal mortality	Intervention study	United States of America, Pakistan	The maternal mortality ratio among Afghan refugees in the area improved from 291 per 1,00,000 live births in 2000 to 102 per 1,00,000 live births in 2004. The proportion of refugee births attended by skilled staff increased from 5% in 1996 to 67% in 2007. Complete prenatal care coverage increased from 49% in 2000 to 90% in 2006, and postnatal coverage more than tripled from 27% in 2000 to 85% in 2006.	Not reported			Afghan refugees located in Afghan refugee settlements in the Hangu district of Khyber Pakhtunkhwa, Pakistan.	Not reported
Knowledge, attitudes and practices of contraception among Afghan refugee women in Pakistan: a cross-sectional study	Raheel et al. (2012) (39)	Karachi	Contraception	Cross-sectional survey	United Arab Emirates, United States of America, Pakistan	Refugee women who are provided subsidized healthcare are more inclined to use contraceptives. It is therefore important that Afghan refugee women living elsewhere in Pakistan be provided with healthcare subsidies, whereby their reproductive health indicators could improve with reduced fertility. We strongly encourage facilities introducing such subsidies to refugees in resource-poor settings to assess the impact through similar inquiry.	650			Afghan refugee women, aged 15–49 years, in Karachi city, who were currently married	Although the study’s results associate healthcare subsidy with better knowledge, attitude, and use of contraceptives, mediators other than the health subsidy could have played a role in this outcome. Since Afghan refugees were in a constant state of influx, an inherent selection bias is inevitable, as those refugee women who went back to Afghanistan might have had different experiences than those who enrolled in the study. The study was also limited to the urban city, therefore generalizability of the findings to the Afghan women refugees in rural parts of Pakistan should be done with caution.
Toward resilient maternal, neonatal and child health care: a qualitative study involving afghan refugee women in Pakistan	Shafiq et al. (2025) (35)	Quetta, Balochistan	Maternal and neonatal mortality	Qualitative study	Pakistan	The study identified significant systemic barriers to accessing MNCH services, such as insufficient funding, inadequate health infrastructure, and discriminatory practices within the healthcare workforce. Additionally, community-level obstacles were prominent, including cultural and language differences, geographical isolation, and economic constraints. The integration of Health-EDRM into local health systems was minimal, with many stakeholders either needing to be made aware of or unengaged with the framework.	20			Afghan refugee women, community elders, and members of the health work force within one of Quetta city's peri-urban squatter settlements	The study did not use formal power analysis to calculate the sample size, which may have missed broader perspectives, particularly variations between urban and rural contexts. There is a potential risk of recall bias as more than 2 years have passed from the initial waves of the COVID-19 pandemic to the time of the interviews. Further, the study's findings, derived from a single UC in Quetta, Balochistan, may not fully represent broader refugee or Pakistani contexts.
The refugees and health crisis: migration policy management and government response to Afghan migrants.	Sumra et al. (2025) (42)	Cities of Islamabad, Quetta, Karachi, and Peshawar	Mental health	Cross sectional quantitative study	Pakistan, China, United Kingdom	The findings show that in the post-pandemic economic crisis, access to health services, relief packages, and risk communication is directly associated with Afghan refugees’ vulnerability (*β* = 0.471, *β* = 0.501, *β* = 0.271 & *β* = 0.259). Notably, the relationship between the post-pandemic economic crisis and Afghan refugees’ vulnerability is mediated by limited access to health services and the unavailability of relief packages. Unavailability of relief packages and lack of risk communication mediate the effect of the refugee crisis on vulnerability. Overall, the proposed model explains 63.3% of the variance in Afghan refugees’ vulnerability with government services. It indicates that Afghan refugees are unable to access relief packages, and there is insufficient communication of risk factors.	429			Afghan refugee families, most of whom were from Peshawar city	The study was restricted to areas populated with the majority of refugees for data collection, which can skew the findings. Convenient sampling is crucial for transparency, but the study used is based on a convenience sample basis due to restricted time and resources. The generalizability of the findings is sufficient because the study focused on refugees’ areas to get a proper response via survey.
Navigating challenges in access to antenatal and intrapartum care: Afghan refugee women's experiences amidst the COVID-19 pandemic in Pakistan	Shafiq et al. (2025) (37)	Quetta city, Balochistan, Pakistan	Maternal and neonatal mortality	Cross-section study	Pakistan, Italy, United States of America	Of 480 MWRAs, only 36.9% sought antenatal care (ANC); only 13.1% received at least four ANC visits. Furthermore, only 38.8% of MWRA had skilled birth attendance. Only 32.9% of MWRAs received at least one ANC and had skilled birth attendance (i.e., comprehensive care). Accessing comprehensive care was associated with maternal age less than 25 years, Tajik ethnicity, and large family size. Predictors of poor access were concerns related to documentation of the refugee women they faced, women with no one at home to accompany them at the health facility, myths and misconceptions related to available care, and the availability of transport. Concerns related to COVID-19 had no association.	480			Afghan married women with at least on child under 2 years old	The cross-sectional design limits inferring causality between identified factors and healthcare-seeking behaviors. This prevents examining changes or trends in behavior over time, and does not provide insights into the causal relationships between variables. The study design also limits the ability to understand the dynamic nature of healthcare access and utilization. One limitation of this study is that the questionnaire used was not a previously validated tool. Additionally, the reliance on self-reported data may introduce recall bias, particularly concerning past healthcare experiences and satisfaction levels.
Leveraging telemedicine to explore contraceptive use and attitudes among refugee women: an observational cross-sectional analysis.	Aga et al. (2025) (38)	Balochistan and Khyber Pakhtunkhwa provinces	Family planning and contraception, maternal and neonatal mortality	Observational cross-sectional study	Pakistan	Refugee women visiting e-health clinics used contraception at a significant rate (68.1%). The majority (71.4%) of women rely on partners for family planning decisions. The primary reasons for using contraception were child spacing (33.2%) and preventing unintended pregnancy (31.1%). Housewives and those with an income of 20,000–40,000 Pakistan rupees (PKR) were more likely to use contraception. Women with limited access to SRH services, as well as those whose spouses make healthcare decisions, were less likely to use them.	576			Women refugees who had attended Sehat Kahani e-health clinics for SRH services	Limited examination of household dynamics and their impact on contraceptive choices. Potential for bias exists due to the reliance on self-reported data. The findings may pertain exclusively to Sehat Kahani and may not comprehensively reflect experiences with alternative telemedicine platforms.

**Figure 2:**
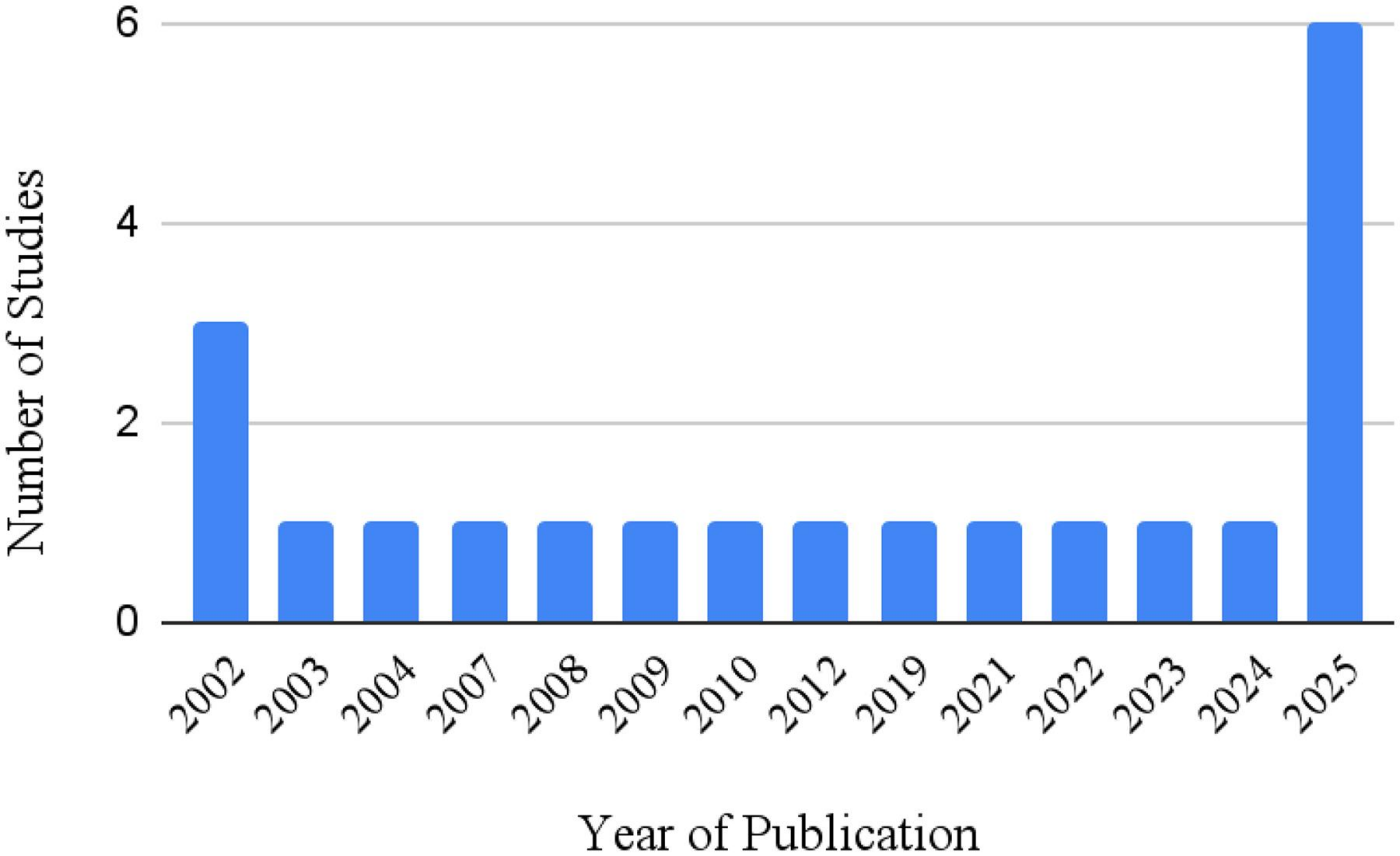
Distribution of publication year of included studies.

There was a clear distinction in the funding and authors' location among the studies. Nine out of 21 studies (43%) were published during or before 2010, demonstrating a heightened interest in Afghan refugee health following the terrorist attack in September 2001, as illustrated in Figure 2 (23–28, 30, 31, 34). Only three of these nine studies were written by authors based in Pakistan (26, 31, 34), while the rest were by authors in the United States. After 2010, 12 studies (57%) were published, all of which were written by authors outside of the USA, including eight from authors in Pakistan (32, 33, 35, 37, 38, 40–42), two from China (11, 29), and one each from the United Arab Emirates (39) and Canada (36). Funding details for the studies published post-2010 were often undisclosed or indicated that they did not receive external funding, and only three studies reported receiving funding (33, 35, 36). Studies that did not mention their funding sources may indicate that there was no dedicated grant for the project.

### 3.2 Quality assessment

Overall, the quality of the included studies was high to medium. All studies met the MMAT's first two screening criteria questions. Of the 21 included studies reviewed using the MMAT, 16 were classified as high quality (4–5 criteria satisfied) (11, 23–30, 33–35, 37–39, 41) and five as medium quality (2–3 criteria satisfied) (31, 36, 40). None of the studies appraised were classified as low quality (0–1 criteria satisfied). One non-randomized study did not meet the qualifications to be assessed using the MMAT because it was not applicable for Question 3.5 (regarding intervention administration, as the study did not administer an intervention) and was excluded from our review (43). Full details of the quality appraisal can be found in the Supplementary Material.

### 3.3 Data themes

**Figure 3:**
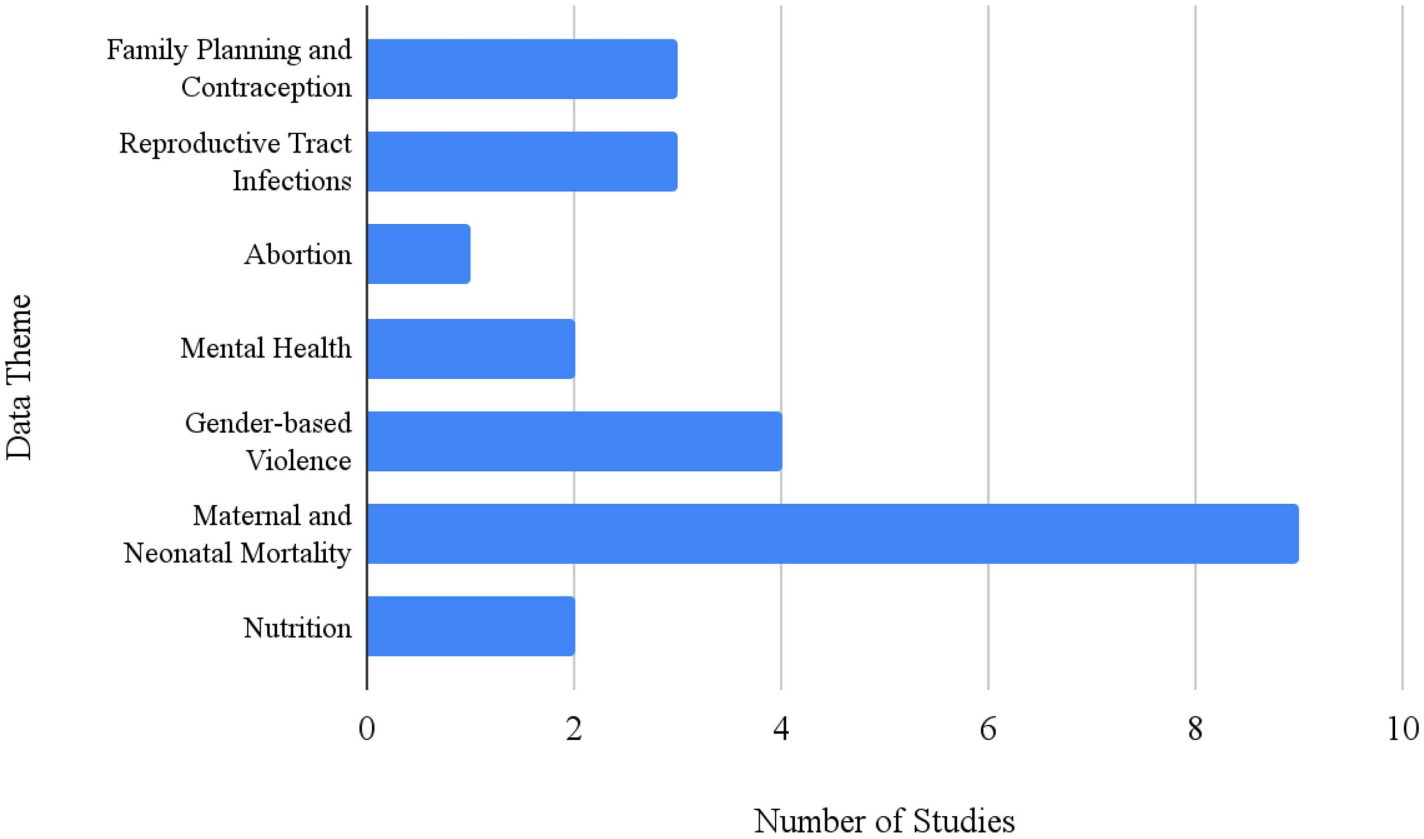
Emerging data themes in the included studies. Studies may focus on multiple data themes.

Nine main themes were identified and broken down into those related to SRH (family planning and contraception, reproductive tract infections, abortion, gender-based violence) and maternal welfare (maternal and neonatal mortality, mental health, nutrition) as demonstrated in Figure 3. Furthermore, cross-cutting themes and comparisons of SRH practices between Afghan and Pakistani mothers are analyzed throughout the articles.

#### 3.3.1 Family planning and contraception

Three studies (14%) examined family planning among Afghan refugees in Pakistan (38, 39, 41). A 2022 study found that 61% of women do not use medical or pharmaceutical contraceptives, and 44% are unaware of their availability (41). A later study that collected data in 2024 reported that 64.4% of women expressed knowledge of at least one contemporary family planning method, and 68.1% were presently using some form of family planning (38).

Of women sampled who use contraceptives in 2022, 52% do so to prevent unplanned pregnancies (41). In 2024, the main reasons for contraceptive use included child spacing (33.2%) and preventing unwanted pregnancies (31.1%) (38). Short-term contraceptive methods are the most commonly used form of modern contraception, with male condoms being among the most common (46%), followed by oral contraceptives (30.7%) and injectables (16.5%) (38). This review did not encounter studies that considered traditional or indigenous methods of contraception.

Seventy-one percent of women reported that their partner has sole authority over decisions regarding family planning, with contraceptive use lower among women whose husbands make exclusive decisions regarding their personal health care. Women who made collaborative decisions with their spouses regarding family planning were also found to have a lower likelihood of using contraception compared to women who made independent, autonomous decisions (38).

In one study, a group of Afghan refugee women was provided with subsidized healthcare from a local non-profit service agency that covered 90% of their medical costs for doctor visits and hospitalizations (39). The subsequent comparison of these Afghan refugees with Afghan refugees without healthcare subsidies found that 90% of the women with subsidized healthcare were aware of family planning options, while only 45% of women who did not receive subsidies had knowledge of these options. Additionally, modern contraceptive use was over twice as high among those with healthcare subsidies, and the likelihood of use increased with age (39). Among subsidized healthcare recipients, tubal ligation was the most common method of contraception (37%), while non-subsidized women predominantly used oral contraceptive pills (40%). Additionally, 89% of women with healthcare subsidies discussed the number of children they should have with their husbands, which was six times more likely than among non-subsidized women (39).

This study also found that recipients of subsidized healthcare were 2.36 times more likely to approve of family planning and 96% less likely to view it as contrary to their religion compared to non-subsidized women (39). The stark differences in contraception use and knowledge between the two groups may be attributed to the higher frequency of interactions with healthcare providers experienced by those with subsidized healthcare. Other contributing factors include the relatively older age of women in the subsidized healthcare group, since contraception use increased with age among women with subsidized healthcare compared to those without (38, 39).

#### 3.3.2 Reproductive tract infections

Three articles (14%) studied reproductive tract infections (RTIs) among Afghan refugee women in Pakistan (30, 32, 40). The most common reproductive tract infection was vaginitis, with 50% of women diagnosed with trichomoniasis, bacterial vaginosis, or candidiasis. Cervicitis was diagnosed in 39.4% of women. Women with vaginal prolapse were significantly more likely to also have vaginitis, with 54.1% of affected women with prolapse also diagnosed with vaginitis (30).

Data collected in 1999 showed that 76.7% of Afghan refugee women visiting Basic Health Units for reproductive health complaints were diagnosed with an RTI (30). A later study, which collected data from 2012 through 2018 by the Commissionerate Afghan Refugees (a Pakistani government agency responsible for overseeing issues related to Afghan refugees in Pakistan), reported an RTI incidence rate of 0.094% (0.94 per 1,000 individuals) among Afghan refugees (32). Additionally, 56.67% of Afghan refugee women were found to have a urinary tract infection (40). Untreated or inadequately treated RTIs can lead to complications such as pelvic inflammatory disease, infertility, ectopic pregnancy, low birth weight (LBW), fetal loss, and an increased risk of HIV transmission (30).

Personal hygiene practices after defecation, sexual intercourse, and menstruation influence the risk of developing RTIs. In a study of 634 Afghan refugee women, the prevalence of suspected pelvic inflammatory disease (PID) was 11.1% among participants using only mud for cleansing after defecation, 18.8% among those using water, and 9.8% among those using old cloth or toilet paper. The use of mud or water, which may get contaminated during the rainy season, is associated with a higher risk of PID (30). Regarding practices for absorbing menstrual blood, the prevalence of PID was 13.3% for women using washcloths, 33.3% for those using no cleansing products, and 26.7% for those using all other methods (30). Further findings of the study indicate 78.5% of women who cleansed by any method after intercourse were diagnosed with an RTI, compared to 55.8% of women who did not cleanse post-intercourse. The most common method of cleansing was the use of an old cloth, reported by 81.2% of women (30).

#### 3.3.3 Abortion

Only one study (5%) examined abortion and abortion outcomes among Afghan refugee women (34). Mothers who have had abortions or miscarriages are at increased risk of anemia. This condition is further exacerbated by high levels of nutritional deficiencies common among Afghan refugees and the increased likelihood of residing in Pakistan's Tribal Districts (44), where anemia rates are elevated (34). There is a strong association between anemia and an increased risk of LBW in infants, highlighting how the compounded risk factors of abortion, nutritional deficiencies, and residence in rural areas negatively impact maternal health (34).

A patient's history of abortions or miscarriages was found to have a detrimental impact on newborn birth weight. Among full-term babies, those whose mothers had a history of abortion/miscarriage were 3.4 times more likely to be small for gestational age compared to full-term babies whose mothers did not have this history (34).

#### 3.3.4 Mental health

Two articles (10%) in this review examined mental health and suicidal feelings among Afghan refugee mothers (31, 42). Thirty-six percent of refugee mothers screened positive for common mental disorders. Of these, 91% also reported having suicidal thoughts in the past month. Additionally, 8% identified suicidal feelings as their primary concern. The high rate of self-reported suicidal feelings in a society where suicide is a social taboo suggests the severity of this issue (31). Maternal mental disorders can lead to emotional, behavioral, and conduct issues in children, and suicidal feelings in primary caregivers are correlated with severe psychopathological outcomes for their children (31). Women also face significant barriers to accessing mental health care due to the ongoing insecurity associated with life in refugee camps (42).

#### 3.3.5 Gender-based violence

This review identified four articles (19%) discussing gender-based violence (GBV) (11, 25, 29, 33). Of the studies covering GBV, one qualitative study interviewed 20 women between the ages of 18 and 50 (25). The second qualitative study included 3 women who ranged in age from 22 to 35 years (29). Participants interviewed in the third qualitative study ranged from 27 to 34 years old (11), and the fourth qualitative study did not provide the ages of participants (33). GBV can result in physical and psychological issues, including chronic pain, mental disorders, STIs, and suicide (25). This violence also harms the physical and mental health of the victims, limits their participation in society, and impedes their access to education (29). Further, the shame, social judgment, and lasting health effects of GBV damage women's employment opportunities and chances for economic independence, increasing feelings of fear and insecurity, and perpetuating a cycle of poverty and dependence (29).

In refugee camp settings, domestic violence is exacerbated by limited resources, poor living conditions, and high rates of unemployment and overcrowding, all of which perpetuate increased family dysfunction. Men may resort to domestic violence as a means to regain a sense of power and control, particularly if they feel they have failed to protect their families (25). Some women justified domestic abuse, asserting that a man has the right to discipline his wife, and they noted that domestic violence is a widely accepted practice (25, 33). The authority systems and Afghan culture's patriarchal norms reinforce traditional gender roles, leaving women without safe spaces and legal protections against GBV, sexual exploitation, and harassment within camp confines (11). Women have reported being subjected to unwanted attention and exploitation by men who often are in control of their access to resources (11).

However, few Afghan refugee women in Pakistan are aware of where to seek support due to unfamiliarity with host country laws, and GBV is not widely recognized on a legal level. Refugee camps also often lack the social services required to protect them from violence, and ultimately, these factors accumulate in very limited reporting (25). Despite these varying challenges, many Afghan refugee women are leaders in refugee camps where they have organized community support networks for resilience and survival (11).

The main cited causes of family conflict include the type of marriage (dowry, number of marriages, exchange marriages), living situation (shared resources and space), and family dynamics (parents-in-law as decision-makers, conflicts among children) (25). Larger dowries are associated with better treatment of wives. “Exchange marriages”, where two families exchange daughters to marry each other's sons, often lead to reciprocal violence between families. Younger brides were found to face more conflict due to inexperience in their marital role (25). Joint family structures, particularly where parents-in-law hold significant decision-making power, also contributed to domestic conflict. For example, conflicts between a mother-in-law and a wife can arise from domestic duties or jealousy over the amount of attention given by the husband (25). As argued by Bartlett, conflict within the family can derive from varying familial relationships, as women “experience violence during day-to-day conflict, and that conflict occurs not only between women and their husbands but also between women and other family members” (23).

Healthcare workers have noted obstacles in addressing GBV due to privacy concerns and sensitivity around family matters, often limited to only treating physical ailments. The lack of standardized protocols or formal training results in inconsistent and insufficient healthcare treatment for GBV victims (25). When violence is reported, providers may discuss with family members about conflict in the home, but these conversations often lack evidence-based guidelines. Severe cases are occasionally referred to community organizations or senior physicians, but it remains unclear how these referrals help victims or how they serve to reduce family violence. The lack of a standardized system to address violence leaves many health workers feeling helpless (25).

#### 3.3.6 Maternal and neonatal mortality

Nine of the 21 articles (42%) included in this review analyzed maternal mortality (23, 24, 26, 27, 29, 35–38), of which two also looked at neonatal mortality (24, 27). The maternal mortality rate (MMR) for Afghan refugee women in Pakistan was 291 per 100,000 live births in 2000, but improved to 102 per 100,000 live births by 2004 (24). Compared to Afghanistan, which had an MMR of 1,900 per 100,000 live births in 2007, Afghan refugee women in Pakistan experience significantly lower maternal mortality rates. This indicates that basic maternal services can substantially reduce mortality rates (23).

Nevertheless, maternal mortality was the leading cause of death among Afghan refugee women of reproductive age, with 41% of deaths attributed to maternal causes. The primary causes included hemorrhage, sepsis, and pregnancy-induced hypertension. It is estimated that 67% of these deaths could have been prevented by avoiding unwanted or mistimed pregnancies, yet only 11% of women who died from maternal causes had used family planning at any point in their lives (23). Eighty-one percent of women who died from maternal-related causes faced barriers to care, including delays in recognizing complications, accessing health facilities, and receiving care at facilities (24). Women who died from maternal causes were often attempting to access healthcare, as evidenced by their deaths occurring en route to or within healthcare facilities (23). The long distances to hospitals and the cost of transportation and treatment often exceeded what families could afford. Additionally, hospitals were frequently overburdened and under-resourced, leading to little confidence in the care provided and a preference for home births (26).

Complete prenatal and postnatal care coverage within 72 h of delivery improved significantly over time, from 49% and 27%, respectively, in 2000 to 90% and 85% in 2006 (24). These improvements were attributed to better access to emergency obstetric care and increased awareness of pregnancy danger signs amongst male family members, who often decide when to seek healthcare, including during pregnancy and childbirth (24). During a similar time period, the percentage of Afghan refugee deliveries in Pakistan attended by skilled personnel also increased, from only 5% in 1996 to 67% by 2007 (24). However, a more recent study, which collected data between 2022 and 2023, reported 38.8% of deliveries had skilled birth attendance (37). About 13% of married Afghan refugee women received at least four antenatal care (ANC) visits, and only 32.9% reported receiving at least one ANC visit and skilled birth attendance, suggesting a gap in comprehensive care during pregnancy (37).

Afghan refugee women in Pakistan also face a high illiteracy rate of 93.33% (40) and a high fertility rate of 4–6 children per woman (38). Together, these can contribute to higher rates of pregnancy complications, a significant contributor to maternal death (23). As argued by Bartlett, “Factors such as high fertility (or number of pregnancies) and low levels of literacy, prevalent in other less-developed countries, were also documented among Afghan refugee women. High fertility increases the chance of pregnancy complications, and low levels of literacy and education are linked to early first births and higher fertility rates” (23).

Private midwifery practices that aim to provide care to Afghan refugees have struggled to remain financially viable, often forcing these businesses to cater to wealthier clients due to the limited financial resources available from refugee populations (35, 36). Midwives catering to Afghan refugees typically earned only 25% of Pakistan's legislated minimum wage, which has led many practices to be forced to close down (36).

The neonatal mortality rate for Afghan refugees in Pakistan was 25 per 1,000 live births in 2000, but by 2006, the neonatal mortality rate had decreased to 20.7 per 1,000 live births. Sixty percent of infants born to Afghan refugees who died from maternal causes were stillborn or died shortly after birth (24).

#### 3.3.7 Nutrition

Two articles (10%) in this review focused on nutrition among Afghan refugees (28, 40). Malnutrition, due to poor dietary intake, is one of the main causes of health complications for Afghan refugee women and can lead to premature deaths of infants and preterm births, as well as perpetuating a cycle where malnourished young women become malnourished mothers (40). Afghan refugees in Pakistan face a high risk of malnutrition, with the annual death rate from undernourishment and poverty being 25 times higher than that from violence.

In a study of 150 Afghan refugee women, 74.7% are underweight, 16.7% are of normal weight, and 8.7% are overweight. Literacy levels may influence body weight, with illiterate individuals more likely to be underweight (40). Many Afghan refugee women also exhibit low hemoglobin levels, indicating low iron deficiency, and have a low body mass index. Additionally, 57.33% of Afghan women present with pale-yellowish skin, suggesting low iron density in the blood due to a poor diet. Hair loss is another sign of malnutrition, as 38% of Afghan refugee women have thin hair, 35% experience extreme hair loss, and 13.33% have dull and dry hair, reflecting deficiencies in protein and essential nutrients. Common dietary deficiencies include a lack of milk and insufficient water intake, with 24% of women skipping dinner daily (40).

For Afghan refugee children aged 6–24 months, good nutritional status was linked to effective nutritional management of childhood illnesses (28). Practices such as increased breastfeeding during illness and recovery, sustained breastfeeding beyond six months, and providing food during and after illness were associated with better growth and nutritional status (28).

#### 3.3.8 Cross-cutting themes

The studies in this review found overlapping barriers for Afghan refugee women accessing healthcare, including financial, sociocultural, and geographic constraints. Over one-third of women experiencing RTIs did not seek treatment, and 93% of low-income refugee mothers did not receive the recommended number of at least four antenatal care visits due to lack of financial resources (30, 37). Due to the insufficient quality of public healthcare, driven by an underfunded and overwhelmed system, Afghan refugees are forced to turn to private healthcare options, leading to high out-of-pocket costs and a considerable financial burden to accessing healthcare (35). Costs related to transportation to hospitals and health care treatment frequently exceed household means, contributing to the barriers women faced who died from maternal-related causes (23, 26, 35, 37).

Sociocultural norms and social stigma have restricted women's autonomy in seeking care and prevented critical and often life-saving conversations regarding SRH topics. Of the 60.4% of women who reported symptoms of prolapse, 29.8% did not seek any medical help due to a lack of cooperation from their husbands/or mother-in-law (30). Restrictive gender norms further inhibit women's ability to seek care by preventing women from accessing care without someone to accompany them (11, 37). Ninety-two percent of women who faced these restrictive practices were unable to seek treatment (37). Documentation requirements also presented a significant barrier as many Afghan refugee women lack necessary legal documents, i.e., registration or identification cards, and are rejected at healthcare facilities (35). About 90% of married women of reproductive age reported not receiving optimal ANC due to challenges related to their legal status (37).

Often, Afghan refugee mothers live in remote regions of Pakistan with limited access to healthcare services and fresh water, where they face long distances and expensive transportation to hospitals (23, 26, 37). Lack of transport availability prevented 74.6% of Afghan women from seeking care (37). Afghan women who died from maternal causes were often attempting to access healthcare, as evidenced by their deaths occurring en route to or within healthcare facilities (23).

According to Shafiq, for Afghan refugee women, there remain “significant systemic barriers to accessing MNCH services, such as insufficient funding, inadequate health infrastructure, and discriminatory practices within the healthcare workforce. Additionally, community-level obstacles were prominent, including cultural and language differences, geographical isolation, and economic constraints” (35).

Due to the low parental literacy rate, both parents face difficulties understanding educational materials and making informed health decisions (35). Regarding pregnancy risk factors, a lack of health knowledge is reported to lead to increased instances of pregnancy loss, including abortion/miscarriage among Afghan refugee mothers (45). Further, language and cultural barriers, such as the availability of female health providers, between Afghan refugees and healthcare providers discourage many women from seeking care due to ineffective communication, cultural misunderstandings, and a mistrust of the health system (35, 42).

#### 3.3.9 Afghan refugee mothers and Pakistani mothers

This systematic review studied the SRH and maternal health outcomes of Afghan refugee women in Pakistan, noting the contrasts between the realities of their counterparts, Pakistani mothers. The findings of this review demonstrate how socio-cultural factors and healthcare access impact maternal health for both groups. Amongst registered Afghan refugees, some have benefited from access to free antenatal care services, including tetanus vaccinations, labor and delivery kits, prenatal counseling, and moral support from female healthcare workers (27). These services have led to improvements in the maternal health of Afghan refugees.

Afghan refugees were found to have a lower infant mortality rate than Pakistanis, at 42 per 1,000 live births compared to 91 per 1,000 (23). Afghan refugee mothers have also demonstrated more effective practices in neonatal care, such as mouth-to-mouth resuscitation, umbilical cord cutting, immediate and exclusive breastfeeding, immunization, and vital registration (27). However, it was more common for Pakistani mothers to involve family members in the birthing process, such as assisting with cutting the umbilical cord and placing the newborn in a safe place upon delivery (27).

Afghan refugee mothers face distinct challenges, including a higher chance of LBW and small gestational age infants compared to Pakistani mothers, and increased incidences of pregnancies ending in an abortion or miscarriage (34, 45). Additionally, Afghan women are more likely to live in the Tribal Districts, come from low-income families, give birth at an older age, and have illiterate husbands. They are less likely to register their pregnancy, further restricting their access to prenatal care and contributing to a higher MMR than their Pakistani counterparts (45). However, both groups demonstrated strengths in exclusive breastfeeding, hypothermia prevention, and paternal involvement in neonatal care (36).

## 4 Discussion

This systematic review aimed to identify gaps in existing research on the SRH and maternal welfare conditions of Afghan refugees in Pakistan. To our knowledge, this is the first systematic review to examine these women's health issues for this demographic as well as delve deeper into the topics of mental health, nutrition, and abortion care. This review fills an important gap in research, given the large number of Afghan refugees both globally and in Pakistan, and the increase in the number of Afghan women refugees in recent years (46). Findings suggest that while the SRH conditions for Afghan refugees in Pakistan have improved over the years covered by this review, significant barriers to accessing healthcare remain, including distance, cost, stigma, and inadequate SRH education. This is further compounded by many social determinants of health Afghan refugees face, such as living in the Tribal Districts and other remote areas that further limit access to health care services (45). Additional research is needed that examines the effects of long-term SRH interventions and how Afghan women in Pakistan exhibit resilience and self-agency in the face of structural oppression, as a limited number of studies were found to examine these concerns.

The present review also found that there is a significant lack of knowledge on abortion care, mental health, mental health services, medical follow-up services, infant care, and maternal nutrition. Furthermore, there is a lack of information on how the Taliban's return to power in August 2021 has impacted SRH and maternal welfare among Afghan refugees in Pakistan.

These findings align with patterns identified in other systematic reviews that have been conducted on the SRH of various refugee populations. One review focused on displaced and migrant women and girls in Uganda, Ethiopia, DR Congo, Somalia, Kenya, Nigeria, Djibouti, Rwanda, and Sierra Leone, highlighting widespread gaps in knowledge and misinformation about contraception and HIV/AIDS, echoing similar findings among Afghan refugee women (47). Across both displaced populations, social norms and cultural stigma were significant barriers to accessing SRH services (47).

Similarly, a review evaluating reproductive health programs in humanitarian settings in sub-Saharan Africa, Asia, and Haiti found that while GBV receives attention in academic research, it remains underrepresented in reproductive health services and program evaluations (48). Similar to the present review, another review on the effectiveness of SRH interventions in humanitarian crises also found zero studies focused on interventions for GBV (49). These reviews and the present review highlight how GBV remains both under-researched and inadequately addressed for Afghan refugees, while in broader humanitarian settings, research on GBV often fails to be translated into implemented interventions (48).

In Uganda, the use of family planning increased with the addition of mobile health outreach and public health centers (47). Notably, the present review found only one study focused on telemedicine, but did not identify any studies implementing mobile health outreach programs. Despite the potential effectiveness of these programs in addressing barriers to care for Afghan mothers, including long distances and travel challenges to reaching care, and the remoteness of the Tribal Districts (38). Indications of the potential effectiveness of such an approach are based on the findings that with more frequent interactions with healthcare providers and expanded education access, there were considerable improvements in SRH outcomes of Afghan refugee women, such as increased contraceptive use (39). In a literacy intervention in a refugee camp in Guinea, 40% of women began using modern family planning methods after exposure to the intervention (50).

However, this present review found only two health intervention studies (10%) to have occurred that focused on SRH, one of which was effective and one was not (24, 36). Given the varied and significant healthcare gaps of Afghan refugee women in Pakistan, there is a clear need for additional intervention studies to address these gaps, including researching how to effectively expand the availability of healthcare education.

Overall, this review highlights the urgent need for improved resources, services, and access within the health sector for Afghan refugees. The review identified several critical areas of need, including expanding health education, addressing barriers to care such as long distances and high costs that make healthcare inaccessible for many Afghan refugees, and improving poor hospital conditions. Many studies found that Afghan refugees cited these factors as reasons for not seeking medical treatment (26, 36, 39). Furthermore, there was a notable lack of studies, with only one study reporting on Afghan refugee women's practices of resilience, agency, and survival, highlighting the active role they play within refugee settings (11). There is a need to develop collaborative, sustainable, and long-term interventions that address barriers to accessing healthcare and expand the availability of health education to Afghan refugees.

### 4.1 Role of conflict on SRH

The Taliban's return to power in Afghanistan in August 2021 further exacerbated the challenges women face in accessing health care due to the dual burden of severe restrictions against women and rapidly deteriorating living conditions (51, 52). Under such restrictive laws in Afghanistan, males continue to dominate the decision-making process for women's health, limiting women's ability to access healthcare. Social norms also require women to obtain permission or financial assistance from their husbands or a male family member regarding health decisions, or be accompanied by a male guardian when leaving their homes, adding further barriers, as women may feel uncomfortable discussing their health with male family members present (52, 53).

This review also found an increased number of publications from 2002 through 2004 (24% of studies), reflecting heightened interest in research on Afghan refugees in the years following 9/11 (23, 26–28, 31). This was followed by a subsequent decrease in publications. Mirroring this heightened interest in research immediately post-9/11 was the amount of aid provided by the United States to Afghan refugees in Pakistan (54). Consistent with research publication trends, aid for Afghan refugees declined rapidly after peaking in the aftermath of the U.S. invasion of Afghanistan in 2001, dropping from $160.47 million in 2002 to $47.1 million in 2005 (54). The parallels between the volume of research and the level of aid for this group demonstrate that interest in this topic is often centered around key world events such as 9/11. As conflicts become protracted and interest wanes, so too does both the aid and funding for research dedicated to better understanding and improving conditions.

Similar to Afghan refugees in Pakistan, there is low SRH knowledge among native Afghans, particularly regarding abortion. One study found that nearly half of Afghan women were unaware of Afghanistan's regulations on abortion, and only 23.4% had a good understanding of abortions (55). The restrictions enacted by the Taliban on women and their health will continue to force more Afghan women into displacement, as evidenced by the recent large influx of Afghan women refugees, resulting in the highest number of refugees recorded in the past 20 years (46).

### 4.2 Limitations of the review

This review has several limitations. The included literature was limited to English-language only due to the author's capacities. We acknowledge that this constraint may exclude literature within scope that is studied in non-English languages and can remediate research gaps found in this systematic review. Despite the exhaustive net cast to retrieve literature from multiple databases, relevant peer-reviewed studies may have been missed, and publication bias may have been introduced by our decision not to search grey literature. Of the relatively small number of studies available for inclusion, it is important to note that a majority of articles focused on the province of Khyber Pakhtunkhwa. Although historically, Khyber Pakhtunkhwa is known for hosting large communities of arriving refugees from Afghanistan in both formal refugee camps as well as informal settlements, this geographic concentration reduces the geographic heterogeneity of included studies and may not reflect the contexts of Afghan refugee women living in other areas of Pakistan (11, 56).

Additionally, there is a lack of research on how political changes, such as the Taliban's reestablishment of control, have impacted the SRH and maternal welfare of Afghan refugees. As well as exploring the long-term effects of interventions on women's health. These gaps in research limit our ability to identify how SRH has been impacted by these issues.

### 4.3 Educational interventions to address SRH and maternal welfare challenges

This review highlights the need for further education programs, which could be crucial tools for improving the maternal welfare of Afghan refugees in Pakistan. Afghan refugees, particularly men, are more likely to be illiterate than their Pakistani counterparts, demonstrating that educational efforts should be tailored to address and accommodate this gap (45). A male family member is often the primary decision maker, especially in dictating women's health needs, and therefore it is vital that information on pregnancy warning signs is accessible, easy to understand, and culturally sensitive (24). Educational programs could include workshops and training sessions that cover essential maternal health topics, reproductive health warning signs, and the importance of SRH services. Education efforts should target not only Afghan men and women but also religious and community leaders to help spread vital information and encourage the use of SRH services.

Additionally, Afghan refugees face significant stigma and difficulties around reporting GBV and locating support, due to a lack of clear policies on the role of healthcare providers and law enforcement officers (25). Education and awareness programs are essential to reducing stigma around GBV and supporting survivors by informing women of their rights, identifying who they can receive support from, decreasing stigma, and building self-esteem. Dedicated efforts on producing more defined policies on GBV are also necessary, as healthcare providers are currently unsure of their role, and police are known to dismiss reports of GBV (25).

## 5 Conclusion

This systematic review identified gaps in the sexual, reproductive, and maternal health of Afghan refugees in Pakistan, exposing under-researched themes such as abortion access, mental health services, and maternal nutrition. Given the current forcible repatriation of Afghan refugees by the Pakistani government, the findings of this review are paramount as the conditions for Afghan refugees and their access to SRH services become increasingly precarious. We found that 67% of maternal deaths were deemed preventable, and 81% of women who died from maternal-related causes faced barriers to accessing care, which severely limited access to SRH services and negatively impacted Afghan refugee women's well-being. These barriers include: cultural norms, barriers to healthcare, and social stigma. Despite these challenges and the structural oppression Afghan women endure, they continue to be active agents within their environments to ensure the survival of themselves and their communities.

A key limitation encountered by this review includes the geographic concentration of our included studies within the province of Khyber Pakhtunkhwa, which reduced the heterogeneity of our findings and limited our ability to conduct an in-depth analysis of the experiences of Afghan refugee women in other areas of Pakistan.

In this review, we articulate several recommendations regarding education, service delivery, policy, and future research based on the evidence identified across the included studies. At the community level, workshops should be developed related to consent and identifying opportunities for support and resources for those experiencing gender-based violence. Interventions and programming should prioritize designing and implementing community-informed and sustainable approaches, including programs that expand health education (particularly tailored towards men and husbands) and further the option of mobile health outreach. The expansion of healthcare education through interventions, such as the use of healthcare subsidies, could have subsequent positive impacts on maternal health by reducing financial barriers to care, facilitating greater healthcare interactions, and ultimately increasing SRH awareness.

Further, policymakers should employ specific community-informed policies focused on expanding access to information and educational materials regarding SRH, and establish comprehensive public transportation options to significantly improve women's ability to access health care and support social and economic independence.

Future research should address the existing gap in information regarding how Afghan women actively engage with and utilize their social and refugee settings to build systems of empowerment, exercise agency, and resist structural oppression. Additionally, further research should also explore how recent and dynamic political changes, such as the Taliban's return to power and the dramatic research funding cuts, have impacted maternal and reproductive health. Key questions remain concerning how to effectively address gaps in mental health and abortion services, in ways that prioritize including the community in the intervention and study design to create sustainable solutions that can address the unique needs of this community.

## Data Availability

The original contributions presented in the study are included in the article/Supplementary Material, further inquiries can be directed to the corresponding author.
