# Supplementary material for: Sexual and reproductive health and maternal welfare among Afghan refugee women in Pakistan: a systematic review
Source: Front Glob Womens Health. 2026 Jul 7;7:1645605. doi: 10.3389/fgwh.2026.1645605 (PMC13384941; doi:10.3389/fgwh.2026.1645605)
Supplement: Supplementary file 1 [file Table1.docx]

Supplementary Material

**Table of Contents**

**Abbreviations**……………………………………………………………………………..…………………………………………….. 2

**Table 3** ……………………………………………………………………………………………………………………………………3

**Preferred Reporting Items for Systematic Reviews and Meta-Analyses (PRISMA) Compliance (Table S1)**………………….…23

**Search Strategy**…………………………………………...……………………………………………………………..……………...31

**Bias Assessments** ………………………………………………………………………………………………….……………………34

**Mixed Methods Appraisal Tool (Table S2, Table S3)**……………………………..………………………………………………….35

**Post Abstract Screening Cuts (Table S4)**…………………………………………………………………….……………………..…39

**Abbreviations**

AIDS: Acquired Immunodeficiency Syndrome

GBV: Gender-Based Violence

HIV: Human Immunodeficiency Virus

MMAT: Mixed Methods Appraisal Tool
MMR: Maternal Mortality Rate
PID: Pelvic Inflammatory Disease

PRISMA: Preferred Reporting Items for Systematic Reviews and Meta-Analyses

RTI: Reproductive Tract Infection
SRH: Sexual and Reproductive Health

STIs: Sexually Transmitted Infections

ANC: Antenatal Care

#

#

#

#

#

#

#

# **Table 3. Details of Included Studies**

**Results**

**Table 3. Details of Included Studies**

| **Study Title** | **Citation** | **Regional Focus and Setting** | **Data Theme** | **Study Design** | **Author(s) Location** | **Key Findings** | **Sample Size (N)** | **Key Predictor** | **OR** | **Population** | **Author Identified Critical Limitations** |
| --- | --- | --- | --- | --- | --- | --- | --- | --- | --- | --- | --- |
|  |  |  |  |  |  |  |  |  |  |  |  |
| Risk factors for low birth weight in the public hospitals at Peshawar, NWFP-Pakistan | Badshah et al. (2008) | Peshawar, Khyber  Pakhtunkhwa | Abortion | Cross-sectional Prospective Survey | Pakistan, United Kingdom | The main geo-demographic risk factors for small for gestational age (SGA) identified in this study, controlling for gestational age of less than 37 weeks, are maternal age, nationality, and consanguinity. Presentation with anemia and the history of previous abortion/miscarriage were also found to be significant independent factors. The adjusted odds ratio for gestational age showed the largest effect in explaining the incidence of low birthweight LBW. The next highest odds ratio was for maternal age below 20 years. The explanatory model included two pairwise interactions, for which the predicted incidence figures for LBW show an increase among the Tribal area with presentation of anemia, and among full-term babies with their mothers having a previous history of abortion/miscarriage. | 1039 | Gestational Age | 6.4 | Single birth mothers from 4 public birth hospitals in Peshawar | Because the study was unable to interview women in privacy, they could not determine induced versus spontaneous abortions. Due to the limited participation of clinicians, the study could not collect information from all mothers admitted for delivery in the four hospitals from August to November. The findings of this study are specific to public hospitals in Khyber Pakhtunkhwa, Pakistan. |
| Human Security and Sustainable Development Goals: The Voices of Afghan Women Refugees in Pakistan | Bakare et al. (2025) | Kohat, Khyber Pakhtunkhwa | Gender-based Violence, | Qualitative study | Pakistan | The key findings suggest that women in camps have cocooned lives, and their patriarchal  cultural set-up perpetuates gender-spatial segregation, which consequently limits women’s opportunities  to access and traverse spaces other than their immediate residential location (camps). In addition, they are  deprived of seeking education outside the radius of the camps and are not allowed to have mobile phones. | 40 | N/A | N/A | Afghan refugee women in refugee camps in Kohat | Not reported |
| Reproductive Tract Disorders among Afghan Refugee Women Attending Health Clinics in Haripur, Pakistan | Balsara et al. (2010) | Haripur,Khyber Pakhtunkhwa | Reproductive Tract Infections | Qualitative Study | United States of America, Pakistan | Over three-fourths (76.7%) of those who reported to the health clinics with reproductive complaints had an RTI. Nearly half (49.5%) of these women were diagnosed with some form of vaginitis, and 14.7% were diagnosed with clinical suspicion of pelvic inflammatory disease (PID). Women with cervical prolapse (p=0.033) or who cleansed after intercourse (p=0.002) were more likely to have vaginitis. There was a significant difference (p=0.017) in the prevalence of suspected PID among women who used mud only (11.1%), any water (18.8%), and an old cloth or toilet paper (9.8%) for cleansing after defecation. | 634 |  |  | Afghan refugee women, aged 12-70 years, living in refugee villages in Haripur, Pakistan, who attended a Basic Health Unit for reproductive health-related complaints. | Selection bias in the enrollment process, as those interviewed were women who self-reported to Basic Health Units for reproductive health complaints. Thus, the results of this study cannot be extrapolated to the Afghan refugee community as a whole. The study also excluded those women who had been on antibiotics. These women may have had more financial resources to seek care. |
| Maternal mortality among Afghan refugees in Pakistan, 1999-2000 | Bartlett et  al. (2002) | Near Hangu in  Khyber Pakhtunkhwa | Gender-based Violence, Maternal and Neonatal Mortality | Population-Based  Retrospective  Cohort Study | United States of America, Pakistan | The census identified 134,406 Afghan refugees and 1197 deaths: a crude mortality rate of 5·5 (95% CI 5·2–5·8) per thousand population. Among the 66 deaths among women of reproductive age, deaths due to maternal causes (n=27) exceeded any other cause (41% [95% CI 29–53]). 16 liveborn and nine stillborn infants were born to women who died of maternal causes; six of the liveborn infants died after birth. Therefore, 60% (15 of 24) of infants born to these women were either born dead or died after birth. Compared with women who died of non-maternal causes, women who died of maternal causes had a greater number of barriers to health care (p=0·001), and their deaths were more likely to be preventable (p<0·05). | 134406 |  |  | 12 Afghan refugee settlements near the village of Hangu, in Khyber Pakhtunkhwa, Pakistan | Not all deaths may have been identified in the census, resulting in lower study mortality estimates. Age and sex-specific mortality rates were not available, making it difficult to assess the overall mortality situation. Misclassification of cause of death during verbal autopsy interviews is possible, since medical records were not available for all women, nor were researchers able to interview health care providers. |
| Nutritional and health status of Afghan refugee women living in Punjab: A cross‐sectional study | Fatima et al. (2023) | Islamabad | Nutrition, Reproductive Tract Infections | Cross-Sectional Study | Pakistan, Iraq | The results indicate the prevalence of underweight, normal weight, and overweight at 74.7%, 16.7%, and 8.7%, respectively. The majority of the women have extremely low hemoglobin (Hb) levels, which indicates iron deficiency as well as low body mass index for their age. The results indicate that there are high chances of severe malnutrition. | 150 |  |  | Afghan refugee women aged 15-30 years, living in sector H-12 of the city of Islamabad, where about 2,000-3,000 Afghan refugees live in muddy houses | Not reported |
| Suicidal feelings run high among mothers in refugee camps: A cross-sectional survey | Hafeez et al. (2003) | Khyber Pakhtunkhwa  Shamshatu and  Shalman refugee camps | Mental Health | Cross-Sectional  Survey | Pakistan | One hundred and six (36%) of women in the sample screened positive for a common mental disorder. Ninety-six (91%) of those screening positive had had suicidal thoughts in the previous month, and nine (8%) rated suicidal feelings as their topmost concern. | 297 |  |  | Afghan refugee mothers with children located in Shamshatu and Shalman refugee camps | Not reported |
| Integrating health care for mothers and children in refugee camps and at district level | Hafeez et al. (2004) | New Jalozai,  New  Shamshatoo,  and Shalman  camps  Case Study 2:  Nowshera and  Gujranwala  districts | Maternal and Neonatal Mortality | Qualitative  Case-Study | Pakistan, United Kingdom | Health care for mothers and children is inadequate in most refugee situations and in poorly resourced countries. The authors argue that, as well as providing primary (home-based) care for basic health care, there is a need to integrate primary care with adequately functioning hospital-based care for a healthcare system to succeed | Not reported |  |  | Afghan refugees located in the refugee camps: New Jalozai, New Shamshatu, and Shalman | Not reported |
| Intimate partner violence among Afghan women living in refugee camps in Pakistan | Hyder et al. (2007) | Khyber Pakhtunkhwa (Jalozai Camp) | Gender-Based Violence | Exploratory Qualitative Study | United States of America | From the interviews, it is evident that women do experience violence during day-to-day conflict, and that conflict occurs not only between women and their husbands but also between women and other family members. It is also clear that health workers have little training or support to deal with cases of violence, and that further exploration of the issues surrounding day-to-day conflict is necessary to develop culturally appropriate interventions. | 20 women of reproductive age and 20 health workers |  |  | Afghan refugees and health workers located in the refugee camp, Jalozai, which is located outside the province's capital city, Peshawar | The study relied on the assistance of health workers for the recruitment of women for the study, and was not able to control for the biases of the health workers during recruitment. |
| Gender dynamics and the role of women in refugee communities in Pakistan: A case study of Afghan refugee camps | Ismail et al. (2025) | Khyber Pakhtunkhwa and Balochistan | Gender-based Violence | Qualitative study | China | The study finds that Afghan women exhibit remarkable agency, participating in informal economic activities, assuming leadership roles within their communities, and advocating for empowerment. The paper identifies key themes of survival strategies, informal support networks, and gendered power dynamics in refugee camps. | 10 |  |  | Afghan refugee women, ministers from local authorities, and NGO staff members | The current study area of Khyber Pakhtunkhwa, along with Balochistan, fails to offer a comprehensive understanding of the wide-ranging experiences of Afghan refugee women across all Pakistani regions and international host destinations. The understanding of Afghan women's experiences in cities and refugee settlements is limited. |
| The reproductive health issues & practices among the Afghan refugee women (with special reference to Afghan Basti in Karachi and Quetta) | Khan et al. (2022) | Karachi and  Quetta | Family Planning and Contraception | Qualitative Study | Pakistan | It was found that many of the women who were experiencing difficulties with their reproductive health also had other health issues, such as diarrhea, gastritis, anemia, respiratory infections, "weakness," renal problems, and skin illnesses. These issues persisted because of poor sanitation and the absence of a functional sewage system in the neighborhood. The Afghan women refugees claim to have mental health problems as well, but they claim they have not been able to access therapists or psychiatrists. | 120 |  |  | Afghan refugee women, aged 15-49 years, living in informal settlements in and around the cities of Quetta and Karachi | Not reported |
| Gender, culture, and migration: A qualitative study of the socioeconomic challenges facing Afghan women refugees in Khyber Pakhtunkhwa, Pakistan | Khan et al. (2024) | Azakhel refugee camp in Noshwera, Khyber Pakhtunkhwa | Gender-Based Violence, Maternal and Neonatal Mortality | Qualitative Study | China, Pakistan | The research highlights challenges from forced migration, gender inequality, cultural norms, and socioeconomic marginalization, causing a holistic crisis for Afghan refugee women in Khyber Pakhtunkhwa, Pakistan. These issues hinder access to education, employment, financial vulnerabilities, and legal uncertainties. | 3 |  |  | Afghan women citizens, aged 22-35 years, in Azakhel refugee camp in Noshwera, Khyber Pakhtunkhwa,  Pakistan. | Not reported |
| Comparison of a positive deviant inquiry with a case-control study to identify factors associated with nutritional status among Afghan refugee children in Pakistan | Lapping et al.  (2002) | Haripur,Khyber Pakhtunkhwa | Nutrition | Qualitative Study | United States of America, Pakistan | The positive deviance inquiry (PDI), identified 12 feeding, caring, and health-seeking behaviors that were not widely practiced. The case-control study (CCS), yielded six significant associations with good nutritional status. Both the PDI and CCS detected feeding behaviors. The PDI alone identified complex phenomena (active feeding and maternal affect). The CCS alone confirmed the beneficial use of health services. | 8 families, 50 children |  |  | Pashtun Afghan refugees living in and around the Haripur refugee camps, which are permanent settlements, two hours northwest of Islamabad | Key limitations include incomplete community verification of the Positive Deviance Inquiry conclusions, potential biomedical biases by those conducting the PDI, inconsistencies amongst the field workers, limited generalizability of specific findings since data was gathered during the summer, and variations among the ages of positive deviant and non-positive deviant children. |
| Disease Status of Afghan Refugees and Migrants in Pakistan | Malik et al. (2019) | Khyber Pakhtunkhwa districts of Peshawar, Dera Ismail Khan, and Bannu | Reproductive Tract Infections | Health Status  Evaluation | United States of America, Pakistan | The most prevalent reported infections were respiratory tract infections (48.05%). Skin diseases and Diarrhea collectively affected 21.08% of Afghan refugees. Overall, the disease burden was higher in females than in males in the Afghan refugee population. | Not reported |  |  | Not reported | Not reported |
| Identification of model newborn care practices through a positive deviance inquiry to guide behavior-change interventions in Haripur, Pakistan | Marsh et al. (2002) | Haripur,Khyber Pakhtunkhwa | Maternal and Neonatal Mortality | Qualitative Study | United Kingdom, United States of America,  Pakistan | The Afghan caregivers showed better use of services and some household practices than their Pakistani counterparts, consistent with the duration of Save the Children Federation/US presence (15 years vs. 18 months, respectively). The practices of both groups for clean delivery, thermal control, immediate and exclusive breastfeeding, and fathers' involvement were weak. But PD individuals, families, and/or birth attendants modeled good maternal care and immediate, routine, and special newborn care. Communities enthusiastically committed to changing behavior and forming neighborhood support groups for better newborn care, including a demand for hygienic delivery. | Not reported |  |  | Afghan refugees located in Camp Five of Basic Health Unit 4, 10 km west of Haripur City | Not reported |
| Midwives providing maternal health services to poor women in the private sector: is it a financially feasible model? | Mumtaz (2021) | Balochistan  (Quetta City) | Maternal and Neonatal Mortality | Qualitative Study | Canada | The single midwife-practices saw a mean of 8.7 ANC patients (range 1-19), attended 2.9 births (range 0-10), and provided care to 1.6 postnatal patients (range 0-7). The average net income of the 11 practices in May 2014 was US$81, but the median was just US$12. To contextualize these incomes, the midwives earned, on average, 25% of Pakistan's minimum monthly living wage. The financial analysis showed that only 3 out of 11 sampled practices could be considered financially viable. The qualitative data revealed that even in practices with reasonable client volumes, the patient's inability to pay was the critical factor in the midwife practices' low net incomes. The research provides empirical evidence of a potential pitfall of private funding models in resource-poor settings where providers rely on impoverished clients to pay user fees. | 11 |  |  | Afghan refugee women trained as midwives in Quetta City, Baluchistan, who were recruited primarily from informal settlements around the city. | Due to an uncertain security situation in the refugee camps in Quetta city and the province of Baluchistan generally, the researchers were unable to have a prolonged presence in the field site. The program also lost contact with many of its midwife alumni, largely because Afghan refugees are a very mobile population. These limitations created limits on follow-up interviews and sample size. |
| Reducing  maternal  mortality among  Afghan refugees  in Pakistan | Purdin et  al. (2009) | Hangu (Khyber Pakhtunkhwa) | Maternal and Neonatal Mortality | Intervention Study | United States of America, Pakistan | The maternal mortality ratio among Afghan refugees in the area improved from 291 per 100000 live births in 2000 to 102 per 100000 live births in 2004. The proportion of refugee births attended by skilled staff increased from 5% in 1996 to 67% in 2007. Complete prenatal care coverage increased from 49% in 2000 to 90% in 2006, and postnatal coverage more than tripled from 27% in 2000 to 85% in 2006. | Not reported |  |  | Afghan refugees located in Afghan refugee settlements in the Hangu district of Khyber Pakhtunkhwa, Pakistan. | Not reported |
| Knowledge, Attitudes and Practices of Contraception among Afghan Refugee Women in Pakistan: A Cross-Sectional Study | Raheel et al. (2012) | Karachi | Contraception | Cross-Sectional Survey | United Arab Emirates, United States of America, Pakistan | Refugee women who are provided subsidized healthcare are more inclined to use contraceptives. It is therefore important that Afghan refugee women living elsewhere in Pakistan be provided with healthcare subsidies, whereby their reproductive health indicators could improve with reduced fertility. We strongly encourage facilities introducing such subsidies to refugees in resource-poor settings to assess the impact through similar inquiry. | 650 |  |  | Afghan refugee women, aged 15-49 years, in Karachi city, who were currently married | Although the study's results associate healthcare subsidy with better knowledge, attitude, and use of contraceptives, mediators other than the health subsidy could have played a role in this outcome. Since Afghan refugees were in a constant state of influx, an inherent selection bias is inevitable, as those refugee women who went back to Afghanistan might have had different experiences than those who enrolled in the study. The study was also limited to the urban city, therefore generalizability of the findings to the Afghan women refugees in rural parts of Pakistan should be done with caution. |
| Toward Resilient Maternal, Neonatal and Child Health Care: A Qualitative Study Involving Afghan Refugee Women in Pakistan | Shafiq et al. (2025) | Quetta, Balochistan | Maternal and Neonatal Mortality | Qualitative study | Pakistan | The study identified significant systemic barriers to accessing MNCH services, such as insufficient funding, inadequate health infrastructure, and discriminatory practices within the healthcare workforce. Additionally, community-level obstacles were prominent, including cultural and language differences, geographical isolation, and economic constraints. The integration of Health-EDRM into local health systems was minimal, with many stakeholders either needing to be made aware of or unengaged with the framework. | 20 |  |  | Afghan refugee women, community elders, and members of the health work force within one of Quetta city’s peri-urban squatter settlements | The study did not use formal power analysis to calculate the sample size, which may have missed broader perspectives, particularly variations between urban and rural contexts. There is a potential risk of recall bias as more than 2 years have passed from the initial waves of the COVID-19 pandemic to the time of the interviews. Further, the study’s findings, derived from a single UC in Quetta, Balochistan, may not fully represent broader refugee or Pakistani contexts. |
| The refugees and health crisis: migration policy management and government response to Afghan migrants. | Sumra et al. (2025) | Cities of Islamabad, Quetta, Karachi, and Peshawar | Mental Health | Cross sectional quantitative study | Pakistan, China, United Kingdom | The findings show that in the post-pandemic economic crisis, access to health services, relief packages, and risk communication is directly associated with Afghan refugees’ vulnerability (β = 0.471, β = 0.501, β = 0.271 & β = 0.259). Notably, the relationship between the post-pandemic economic crisis and Afghan refugees’ vulnerability is mediated by limited access to health services and the unavailability of relief packages. Unavailability of relief packages and lack of risk communication mediate the effect of the refugee crisis on vulnerability. Overall, the proposed model explains 63.3% of the variance in Afghan refugees’ vulnerability with government services. It indicates that Afghan refugees are unable to access relief packages, and there is insufficient communication of risk factors. | 429 |  |  | Afghan refugee families, most of whom were from Peshawar city | The study was restricted to areas populated with the majority of refugees for data collection, which can skew the findings. Convenient sampling is crucial for transparency, but the study used is based on a convenience sample basis due to restricted time and resources. The generalizability of the findings is sufficient because the study focused on refugees’ areas to get a proper response via survey. |
| Navigating challenges in access to antenatal and intrapartum care: Afghan refugee women’s experiences amidst the COVID-19 pandemic in Pakistan | Shafiq et al. (2025) | Quetta city, Balochistan, Pakistan | Maternal and Neonatal Mortality | Cross-section study | Pakistan, Italy, United States of America | Of 480 MWRAs, only 36.9% sought antenatal care (ANC); only 13.1% received at least four ANC visits. Furthermore, only 38.8% of MWRA had skilled birth attendance. Only 32.9% of MWRAs received at least one ANC and had skilled birth attendance (i.e., comprehensive care). Accessing comprehensive care was associated with maternal age less than 25 years, Tajik ethnicity, and large family size. Predictors of poor access were concerns related to documentation of the refugee women they faced, women with no one at home to accompany them at the health facility, myths and misconceptions related to available care, and the availability of transport. Concerns related to COVID-19 had no association. | 480 |  |  | Afghan married women with at least on child under 2 yrs old | The cross-sectional design limits inferring causality between identified factors and healthcare-seeking behaviors. This prevents examining changes or trends in behavior over time, and does not provide insights into the causal relationships between variables. The study design also limits the ability to understand the dynamic nature of healthcare access and utilization. One limitation of this study is that the questionnaire used was not a previously validated tool. Additionally, the reliance on self-reported data may introduce recall bias, particularly concerning past healthcare experiences and satisfaction levels. |
| Leveraging telemedicine to explore contraceptive use and attitudes among refugee women: an observational cross-sectional analysis. | Zafar Aga et al. (2025) | Balochistan and Khyber Pakhtunkhwa provinces | Family Planning and Contraception, Maternal and Neonatal Mortality | Observational cross-sectional study | Pakistan | Refugee women visiting e-health clinics used contraception at a significant rate (68.1%). The majority (71.4%) of women rely on partners for family planning decisions. The primary reasons for using contraception were child spacing (33.2%) and preventing unintended pregnancy (31.1%). Housewives and those with an income of 20 000-40 000 Pakistan rupees (PKR) were more likely to use contraception. Women with limited access to SRH services, as well as those whose spouses make healthcare decisions, were less likely to use them. | 576 |  |  | Women refugees who had attended Sehat Kahani e-health clinics for SRH services | Limited examination of household dynamics and their impact on contraceptive choices. Potential for bias exists due to the reliance on self-reported data. The findings may pertain exclusively to Sehat Kahani and may not comprehensively reflect experiences with alternative telemedicine platforms. |
|  |  |  |  |  |  |  |  |  |  |  |  |
|  |  |  |  |  |  |  |  |  |  |  |  |
|  |  |  |  |  |  |  |  |  |  |  |  |
|  |  |  |  |  |  |  |  |  |  |  |  |

# **PRISMA Compliance**

This study complies with the [PRISMA 2020 recommendations](http://www.prisma-statement.org/). The table below shows the completed checklist and the locations in which the respective items can be found within the paper.

**Supplementary Table 1.** PRISMA 2020 Checklist

| **Section and Topic** | **Item #** | **Checklist item** | **Location where item is reported** |
| --- | --- | --- | --- |
| **TITLE** | | |  |
| Title | 1 | Identify the report as a systematic review. | Paper title |
| **ABSTRACT** | | |  |
| Abstract | 2 | See the PRISMA 2020 for Abstracts checklist. | N/A |
| **INTRODUCTION** | | |  |
| Rationale | 3 | Describe the rationale for the review in the context of existing knowledge. | Main text Introduction, pg.4 |
| Objectives | 4 | Provide an explicit statement of the objective(s) or question(s) the review addresses. | Main text Introduction, pg.5 |
| **METHODS** | | |  |
| Eligibility criteria | 5 | Specify the inclusion and exclusion criteria for the review and how studies were grouped for the syntheses. | Table 1 and Main text Methods Section 2.2, “Search Strategy and Selection Criteria,” pg. 6 |
| Information sources | 6 | Specify all databases, registers, websites, organisations, reference lists and other sources searched or consulted to identify studies. Specify the date when each source was last searched or consulted. | Main text Methods Section 2.2, “Search Strategy and Selection Criteria,” pg. 6 |

| Search strategy | 7 | Present the full search strategies for all databases, registers and websites, including any filters and limits used. | Supplementary Material, “Search Strategy “pgs. 27-30 |
| --- | --- | --- | --- |
| Selection process | 8 | Specify the methods used to decide whether a study met the inclusion criteria of the review, including how many reviewers screened each record and each report retrieved, whether they worked independently, and if applicable, details of automation tools used in the process. | Main text Methods Section 2.3, “Screening and Data Extraction,” pg. 8 |
| Data collection process | 9 | Specify the methods used to collect data from reports, including how many reviewers collected data from each report, whether they worked independently, any processes for obtaining or confirming data from study investigators, and if applicable, details of automation tools used in the process. | Main text Methods Section 2.3, “Screening and Data Extraction,” pg. 8 |
| Data items | 10a | List and define all outcomes for which data were sought. Specify whether all results that were compatible with each outcome domain in each study were sought (e.g. for all measures, time points, analyses), and if not, the methods used to decide which results to collect. | Main text Methods Section 2.3 “Screening and Data Extraction,” pg. 8 |
|  | 10b | List and define all other variables for which data were sought (e.g. participant and intervention characteristics, funding sources). Describe any assumptions made about any missing or unclear information. | Main text Methods Section 2.3 “Screening and Data Extraction,”, pg. 8 |
| Study risk of bias assessment | 11 | Specify the methods used to assess risk of bias in the included studies, including details of the tool(s) used, how many reviewers assessed each study and whether they worked independently, and if applicable, details of automation tools used in the process. | Main text Methods Section 2.4, “Quality Assessment,” pgs. 9-10 |
| Effect measures | 12 | Specify for each outcome the effect measure(s) (e.g. risk ratio, mean difference) used in the synthesis or presentation of results. | N/A |
| Synthesis methods | 13a | Describe the processes used to decide which studies were eligible for each synthesis (e.g. tabulating the study intervention characteristics and comparing against the planned groups for each synthesis (item #5)). | N/A |
|  | 13b | Describe any methods required to prepare the data for presentation or synthesis, such as handling of missing summary statistics, or data conversions. | N/A |
|  | 13c | Describe any methods used to tabulate or visually display results of individual studies and syntheses. | Main text Methods Section 2.3, “Screening and Data Extraction,” pg. 8 |
|  | 13d | Describe any methods used to synthesize results and provide a rationale for the choice(s). If meta-analysis was performed, describe the model(s), method(s) to identify the presence and extent of statistical heterogeneity, and software package(s) used. | Main text Methods Section 2.3, “Screening and Data Extraction,” pg. 9 |
|  | 13e | Describe any methods used to explore possible causes of heterogeneity among study results (e.g. subgroup analysis, meta-regression). | Main text Methods Section 2.3, “Screening and Data Extraction,” pg. 9 |
|  | 13f | Describe any sensitivity analyses conducted to assess robustness of the synthesized results. | Main text Methods Section 2.3, “Screening and Data Extraction,” pg. 9 |

| Reporting bias assessment | 14 | Describe any methods used to assess risk of bias due to missing results in a synthesis (arising from reporting biases). | Supplementary Material, “Bias Assessments,” pg. 31 |
| --- | --- | --- | --- |
| Certainty assessment | 15 | Describe any methods used to assess certainty (or confidence) in the body of evidence for an outcome. | N/A |
| **RESULTS** | | |  |
| Study selection | 16a | Describe the results of the search and selection process, from the number of records identified in the search to the number of studies included in the review, ideally using a flow diagram. | Main text Results Section 3.1 “Article Characteristics,” pg. 10 |
|  | 16b | Cite studies that might appear to meet the inclusion criteria, but which were excluded, and explain why they were excluded. | Supplementary Material, “Excluded Study Characteristics,” pgs. 38-40 |
| Study characteristics | 17 | Cite each included study and present its characteristics. | Main text Results Section 3.1, “Article Characteristics,” pg. 10 |
| Risk of bias in studies | 18 | Present assessments of risk of bias for each included study. | Supplementary Material, “Bias Assessments” pg. 31 |
| Results of individual studies | 19 | For all outcomes, present, for each study: (a) summary statistics for each group (where appropriate) and (b) an effect estimate and its precision (e.g. confidence/credible interval), ideally using structured tables or plots. | Main text Results Section 3.2, “Data Themes” Table 3 |
| Results of syntheses | 20a | For each synthesis, briefly summarise the characteristics and risk of bias among contributing studies. | Supplementary Material, “Bias Assessments”, pg. 31 |
|  | 20b | Present results of all statistical syntheses conducted. If meta-analysis was done, present for each the summary estimate and its precision (e.g. confidence/credible interval) and measures of statistical heterogeneity. If comparing groups, describe the direction of the effect. | N/A |
|  | 20c | Present results of all investigations of possible causes of heterogeneity among study results. | N/A |
|  | 20d | Present results of all sensitivity analyses conducted to assess the robustness of the synthesized results. | N/A |
| Reporting biases | 21 | Present assessments of risk of bias due to missing results (arising from reporting biases) for each synthesis assessed. | N/A |
| Certainty of evidence | 22 | Present assessments of certainty (or confidence) in the body of evidence for each outcome assessed. | N/A |
| **DISCUSSION** | | |  |
| Discussion | 23a | Provide a general interpretation of the results in the context of other evidence. | Main text Discussion, pgs. 21- 24 |
|  | 23b | Discuss any limitations of the evidence included in the review. | Main text Discussion section 4.2 “Limitations of the Review,” pgs. 24-25 |
|  | 23c | Discuss any limitations of the review processes used. | Main text Discussion section 4.2 “Limitations of the Review,” pgs. 24-25 |
|  | 23d | Discuss implications of the results for practice, policy, and future research. | Main text Conclusion pgs. 25-26 |
| **OTHER INFORMATION** | | |  |
| Registration and protocol | 24a | Provide registration information for the review, including register name and registration number, or state that the review was not registered. | Main text, Methods Section 2.1, “Review Focus and Guidelines,” pg. 6 |
|  | 24b | Indicate where the review protocol can be accessed, or state that a protocol was not prepared. | Main text Methods Section 2.2 “Search Strategy and Selection Criteria,” pg. 6 |
|  | 24c | Describe and explain any amendments to information provided at registration or in the protocol. | N/A |
| Support | 25 | Describe sources of financial or non-financial support for the review, and the role of the funders or sponsors in the review. | Main text. “Funding section,” pg. 26 |
| Competing interests | 26 | Declare any competing interests of review authors. | Main text, “Competing interests,” pg. 26 |
| Availability of data, code and other materials | 27 | Report which of the following are publicly available and where they can be found: template data collection forms; data extracted from included studies; data used for all analyses; analytic code; any other materials used in the review. | Main text, “Data Sharing,” pg. 26 |

#

# **Search Strategy**

*Literature Search*

We used electronic bibliographic databases for peer-reviewed publications that were searched using a list of key terms and compiled through Rayyan. The rest of the protocol was followed throughout the review process.

*Electronic Bibliographic Databases*

Initial database searches were conducted in September 2024 and August 19, 2024, and subsequently updated on January 22, 2026, to ensure the most current research is presented in the review. For the updated searches, database-specific date limits were applied as consistently as possible between August 19, 2024, and December 31, 2025. The following databases were searched: Boston University Online Library Database, PubMed, Science Direct, Web of Science, Embase, and EBSCOhost. Each database search was optimized for comprehensive literature retrieval associated with sexual, reproductive, or maternal health. When available, filters for female populations and peer-reviewed articles were applied.

Search query per database is available below.

1. **Science Direct**

(Afghan AND refugee) AND (women OR female) AND (“reproductive health” OR “maternal health”) AND Pakistan

1. **EBSCOHost**

Search mode: Proximity Search, Turn off Apply related words, Also search within the full text of the articles, and Apply Equivalent Subjects

(Afghan* AND “Afghan refugee*” OR “Afghan migrant*” OR “Afghan refugee women” OR migrant* OR refugee* OR “refugee women” OR asylum OR displaced OR “displaced person*”) AND (adolescence* OR adolescent* OR female OR girl* OR teen* OR teenager* OR woman OR women OR “young female*” OR “young marriage” OR “young person” OR “young women” OR youth) AND ( “access to maternal healthcare” OR AIDS OR antenatal OR “antenatal care” OR birth OR “birth complications” OR “birth outcomes” OR “child marriage” OR condom* OR contraception* OR “contraceptive use” OR “dowry death” OR “early marriage” OR “early motherhood” OR “family health” OR “family planning” OR “forced marriage” OR “forced sex” OR “health outcomes” OR HIV OR maternal OR “maternal health” OR “maternal healthcare access” OR “maternal mortality” OR “maternal nutrition” OR “maternal well-being” OR “menstrual hygiene” OR menstruation OR “mental health” OR “mortality rate” OR motherhood OR “neonatal mortality” OR nutrition OR “obstetric care” OR “perinatal outcomes” OR “physical relationship” OR post-natal OR “post-natal care” OR pregnancy OR “pregnancy complications” OR rape OR relationship OR reproductive OR “reproductive health” OR “reproductive health care” OR “reproductive health issues” OR “reproductive morbidity” OR “reproductive rights” OR “safe childbirth” OR “sexual activity” OR sex OR “sex education” OR sexual OR “sexual abuses” OR “sexual assault” OR “sexual behavior” OR “sexual coercion” OR “sexual experience” OR “sexual health” OR “sexual initiation” OR “sexual intercourse” OR “skilled birth attendants” OR suicide OR “teenage pregnancy” OR violence OR “womans health ) AND (Pakistan)

1. **PubMed**

( Afghan[tiab] AND “Afghan refugee*”[tiab] OR “Afghan migrant*”[tiab] OR “Afghan refugee women”[tiab] OR migrant*[tiab] OR refugee*[Mesh] OR “refugee women”[tiab] OR asylum[tiab] OR displaced[tiab] OR “displaced person*”[tiab] ) AND ( adolescence*[tiab] OR adolescent*[tiab] OR female[Mesh] OR girl*[tiab] OR teen*[tiab] OR teenager*[tiab] OR woman[tiab] OR women[Mesh] OR “young female*”[tiab] OR “young marriage”[tiab] OR “young person”[tiab] OR “young women”[tiab] OR youth[tiab] ) AND (“access to maternal healthcare”[tiab] OR AIDS[tiab] OR antenatal[tiab] OR “antenatal care”[tiab] OR birth[tiab] OR “birth complications”[tiab] OR “birth outcomes”[tiab] OR “child marriage”[tiab] OR condom*[tiab] OR contraception*[tiab] OR “contraceptive use”[tiab] OR “dowry death”[tiab] OR “early marriage”[tiab] OR “early motherhood”[tiab] OR “family health”[tiab] OR “family planning”[tiab] OR “forced marriage”[tiab] OR “forced sex”[tiab] OR “health outcomes”[tiab] OR HIV[tiab] OR maternal[tiab] OR “maternal health”[Mesh] OR “maternal healthcare access”[tiab] OR “maternal mortality”[tiab] OR “maternal nutrition”[tiab] OR “maternal well-being”[tiab] OR “menstrual hygiene”[tiab] OR menstruation[tiab] OR “mental health”[tiab] OR “mortality rate”[tiab] OR motherhood[tiab] OR “neonatal mortality”[tiab] OR nutrition[tiab] OR “obstetric care”[tiab] OR “perinatal outcomes”[tiab] OR “physical relationship”[tiab] OR post-natal[tiab] OR “post-natal care”[tiab] OR pregnancy[tiab] OR “pregnancy complications”[tiab] OR rape[tiab] OR relationship[tiab] OR reproductive[tiab] OR “reproductive health”[Mesh] OR “reproductive health care”[tiab] OR “reproductive health issues”[tiab] OR “reproductive morbidity”[tiab] OR “reproductive rights”[tiab] OR “safe childbirth”[tiab] OR “sexual activity”[tiab] OR sex[tiab] OR “sex education”[tiab] OR sexual[tiab] OR “sexual abuses”[tiab] OR “sexual assault”[tiab] OR “sexual behavior”[tiab] OR “sexual coercion”[tiab] OR “sexual experience”[tiab] OR “sexual health”[tiab] OR “sexual initiation”[tiab] OR “sexual intercourse”[tiab] OR “skilled birth attendants”[tiab] OR suicide[tiab] OR “teenage pregnancy”[tiab] OR violence[tiab] OR “womans health”[tiab] ) AND (Pakistan[Mesh])

1. **Boston University Online Library Database**

(Afghan* AND “Afghan refugee” OR Afghan migrant*” OR “Afghan refugee women” OR migrant* OR refugee* OR “refugee women” OR asylum OR displaced OR “displaced person*”) AND (adolescence* OR adolescent* OR female OR girl* OR teen* OR teenager* OR woman OR women OR “young female*” OR “young marriage” OR “young person” OR “young women” OR youth) AND ( “access to maternal healthcare” OR AIDS OR antenatal OR “antenatal care” OR birth OR “birth complications” OR “birth outcomes” OR “child marriage” OR condom* OR contraception* OR “contraceptive use” OR “dowry death” OR “early marriage” OR “early motherhood” OR “family health” OR “family planning” OR “forced marriage” OR “forced sex” OR “health outcomes” OR HIV OR maternal OR “maternal health” OR “maternal healthcare access” OR “maternal mortality” OR “maternal nutrition” OR “maternal well-being” OR “menstrual hygiene” OR menstruation OR “mental health” OR “mortality rate” OR motherhood OR “neonatal mortality” OR nutrition OR “obstetric care” OR “perinatal outcomes” OR “physical relationship” OR post-natal OR “post-natal care” OR pregnancy OR “pregnancy complications” OR rape OR relationship OR reproductive OR “reproductive health” OR “reproductive health care” OR “reproductive health issues” OR “reproductive morbidity” OR “reproductive rights” OR “safe childbirth” OR “sexual activity” OR sex OR “sex education” OR sexual OR “sexual abuses” OR “sexual assault” OR “sexual behavior” OR “sexual coercion” OR “sexual experience” OR “sexual health” OR “sexual initiation” OR “sexual intercourse” OR “skilled birth attendants” OR suicide OR “teenage pregnancy” OR violence OR “womans health” ) AND (Pakistan)

1. **Web of Science**

Query 1

(Afghan* AND “Afghan refugee” OR Afghan migrant*” OR “Afghan refugee women” OR migrant* OR refugee* OR “refugee women” OR asylum OR displaced OR “displaced person*” ) AND ( adolescence* OR adolescent* OR female OR girl* OR teen* OR teenager* OR woman OR women OR “young female*” OR “young marriage” OR “young person” OR “young women” OR youth ) AND ( “access to maternal healthcare” OR AIDS OR antenatal OR “antenatal care” OR birth OR “birth complications” OR “birth outcomes” OR “child marriage” OR condom* OR contraception* OR “contraceptive use” OR “dowry death” OR “early marriage” OR “early motherhood” OR “family health” OR “family planning” OR “forced marriage” OR “forced sex” OR “health outcomes” OR HIV OR maternal OR “maternal health” OR “maternal healthcare access” OR “maternal mortality” OR “maternal nutrition” OR “maternal well-being” OR “menstrual hygiene” OR menstruation OR “mental health” OR “mortality rate” OR motherhood OR “neonatal mortality” OR nutrition OR “obstetric care” OR “perinatal outcomes” OR “physical relationship” OR post-natal OR “post-natal care” OR pregnancy OR “pregnancy complications” OR rape OR relationship OR reproductive OR “reproductive health” OR “reproductive health care” OR “reproductive health issues” OR “reproductive morbidity” OR “reproductive rights” OR “safe childbirth” OR “sexual activity” OR sex OR “sex education” OR sexual OR “sexual abuses” OR “sexual assault” OR “sexual behavior” OR “sexual coercion” OR “sexual experience” OR “sexual health” OR “sexual initiation” OR “sexual intercourse” OR “skilled birth attendants” OR suicide OR “teenage pregnancy” OR violence OR “womans health” ) AND ( Pakistan )

1. **Embase**

(afghan*:ti,ab,kw AND 'afghan refugee':ti,ab,kw OR 'afghan migrant*':ti,ab,kw OR 'afghan refugee women':ti,ab,kw OR migrant*:ti,ab,kw OR refugee*:ti,ab,kw OR 'refugee women':ti,ab,kw OR asylum:ti,ab,kw OR displaced:ti,ab,kw OR 'displaced person*':ti,ab,kw) AND (adolescence*:ti,ab,kw OR adolescent*:ti,ab,kw OR female:ti,ab,kw OR girl*:ti,ab,kw OR teen*:ti,ab,kw OR teenager*:ti,ab,kw OR woman:ti,ab,kw OR women:ti,ab,kw OR 'young female*':ti,ab,kw OR 'young marriage':ti,ab,kw OR 'young person':ti,ab,kw OR 'young women':ti,ab,kw OR youth:ti,ab,kw) AND ('access to maternal healthcare':ti,ab,kw OR aids:ti,ab,kw OR antenatal:ti,ab,kw OR 'antenatal care':ti,ab,kw OR birth:ti,ab,kw OR 'birth complications':ti,ab,kw OR 'birth outcomes':ti,ab,kw OR 'child marriage':ti,ab,kw OR condom*:ti,ab,kw OR contraception*:ti,ab,kw OR 'contraceptive use':ti,ab,kw OR 'dowry death':ti,ab,kw OR 'early marriage':ti,ab,kw OR 'early motherhood':ti,ab,kw OR 'family health':ti,ab,kw OR 'family planning':ti,ab,kw OR 'forced marriage':ti,ab,kw OR 'forced sex':ti,ab,kw OR 'health outcomes':ti,ab,kw OR hiv:ti,ab,kw OR maternal:ti,ab,kw OR 'maternal health':ti,ab,kw OR 'maternal healthcare access':ti,ab,kw OR 'maternal mortality':ti,ab,kw OR ‘maternal nutrition':ti,ab,kw OR 'maternal well-being':ti,ab,kw OR 'menstrual hygiene':ti,ab,kw OR menstruation:ti,ab,kw OR 'mental health':ti,ab,kw OR 'mortality rate':ti,ab,kw OR motherhood:ti,ab,kw OR 'neonatal mortality':ti,ab,kw OR nutrition:ti,ab,kw OR 'obstetric care':ti,ab,kw OR 'perinatal outcomes':ti,ab,kw OR 'physical relationship':ti,ab,kw OR 'post natal':ti,ab,kw OR 'post-natal care':ti,ab,kw OR pregnancy:ti,ab,kw OR 'pregnancy complications':ti,ab,kw OR rape:ti,ab,kw OR relationship:ti,ab,kw OR reproductive:ti,ab,kw OR 'reproductive health':ti,ab,kw OR 'reproductive health care':ti,ab,kw OR 'reproductive health issues':ti,ab,kw OR 'reproductive morbidity':ti,ab,kw OR 'reproductive rights':ti,ab,kw OR 'safe childbirth':ti,ab,kw OR 'sexual activity':ti,ab,kw OR sex:ti,ab,kw OR 'sex education':ti,ab,kw OR sexual:ti,ab,kw OR 'sexual abuses':ti,ab,kw OR 'sexual assault':ti,ab,kw OR 'sexual behavior':ti,ab,kw OR 'sexual coercion':ti,ab,kw OR 'sexual experience':ti,ab,kw OR 'sexual health':ti,ab,kw OR 'sexual initiation':ti,ab,kw OR 'sexual intercourse':ti,ab,kw OR 'skilled birth attendants':ti,ab,kw OR suicide:ti,ab,kw OR 'teenage pregnancy':ti,ab,kw OR violence:ti,ab,kw OR 'womans health':ti,ab,kw) AND pakistan:ti,ab,kw

**Bias Assessments**

*Study Risk of Bias*

As detailed in the main text, we assessed the studies’ risk of bias using the Mixed Methods Appraisal Tool (MMAT). The MMAT tool can be used for five study designs: qualitative research, randomized controlled trials, non-randomized studies, quantitative descriptive studies, and mixed methods studies. Following initial screening, articles are categorized based on article type and screened with their respective questions. MMAT discourages reviewers from creating an overall score.

# **Mixed Methods Appraisal Tool (MMAT) Scoring**

**Supplementary Table 4**

*Mixed Methods Appraisal Tool (MMAT), version 2018*

| **Category of study designs** | **Methodological quality criteria** | **Responses** | | | | | | |
| --- | --- | --- | --- | --- | --- | --- | --- | --- |
|  |  |  | |  |  | |  | |
| Screening questions  (for all study types) | S1: Are there clear research questions? | **Yes No Can’t tell Comments** | | | | | | |
|  | S2: Do the collected data allow to answer the research question? |  | | | | | | |
| Qualitative | 1.1. Is the qualitative approach appropriate to answer the research question? |  | | | | | | |
|  | 1.2. Are the qualitative data collection methods adequate to address the research question? |  | | | | | | |
|  | 1.3. Are the findings adequately derived from the data? |  | | | | | | |
|  | 1.4. Is the interpretation of results sufficiently substantiated by data? |  | | | | | | |
|  | 1.5. Is there coherence between qualitative data sources, collection, analysis and interpretation? |  | | | | | | |
| Quantitative descriptive | 4.1. Is the sampling strategy relevant to address the research question? |  | | | | | | |
|  | 4.2. Is the sample representative of the target population? |  | | | | | | |
|  | 4.3. Are the measurements appropriate? |  | | | | | | |
|  | 4.4. Is the risk of nonresponse bias low? |  | | | | | | |
|  | 4.5. Is the statistical analysis appropriate to answer the research question? |  | | | | | | |
|  |  | *continued* | | | | | | |
| **Category of study designs** | **Methodological quality criteria** | **Responses** | | | | | | |
|  |  | **Yes** | **No** | | | **Can’t tell** | | **Comments** |
| Quantitative non-randomized | 3.1. Are the participants representative of the target population? |  | | | | | | |
|  | 3.2. Are measurements appropriate regarding both the outcome and exposure/intervention? |  | | | | | | |
|  | 3.3. Are there complete outcome data? |  | | | | | | |
|  | 3.4. Are the confounders accounted for in the design and analysis? |  | | | | | | |
|  | 3.5. During the study period, is the intervention/exposure administered as intended? |  | | | | | | |
| Mixed methods | 5.1. Is there an adequate rationale for using a mixed methods design to address the research question? |  | | | | | | |
|  | 5.2. Are the different components of the study effectively integrated to answer the research question? |  | | | | | | |
|  | 5.3. Are the outputs of the integration of qualitative and quantitative components adequately interpreted? |  | | | | | | |
|  | 5.4. Are divergences and inconsistencies between quantitative and qualitative results adequately addressed? |  | | | | | | |
|  | 5.5. Do the different components of the study adhere to the quality criteria of each tradition of the methods involved? |  | | | | | | |

**Supplementary Table 5**

*Quality Assessment using the Mixed-Methods Assessment Tool*

Note: Y = Yes, N = No, U = Can’t tell

| **Citation** | **Screening** | | **Qualitative** | | | | | **Quantitative non-randomized** | | | | | **Quantitative Descriptive** | | | | | **Mixed Methods** | | | | | | | **MMAT Scoring** |
| --- | --- | --- | --- | --- | --- | --- | --- | --- | --- | --- | --- | --- | --- | --- | --- | --- | --- | --- | --- | --- | --- | --- | --- | --- | --- |
|  | **S1** | **S2** | **1.1** | **1.2** | **1.3** | **1.4** | **1.5** | **3.1** | **3.2** | **3.3** | **3.4** | **3.5** | **4.1** | **4.2** | **4.3** | **4.4** | **4.5** | **5.1** | **5.2** | **5.3** | **5.4** | | **5.5** | |  |
| Bakare et al. (2025) | Y | Y | Y | Y | Y | Y | y |  |  |  |  |  |  |  |  |  |  |  |  |  | |  | |  | High |
| Balsara et al. (2010) | Y | Y |  |  |  |  |  |  |  |  |  |  | Y | Y | Y | Y | Y |  |  |  | |  | |  | High |
| Bartlett et  al. (2002) | Y | Y |  |  |  |  |  |  |  |  |  |  | Y | Y | Y | Y | Y |  |  |  | |  | |  | High |
| Fatima et al. (2023) | Y | Y |  |  |  |  |  |  |  |  |  |  | Y | N | Y | N | Y |  |  |  | |  | |  | Moderate |
| Hafeez et al. (2003) | Y | Y |  |  |  |  |  |  |  |  |  |  | Y | N | Y | N | Y |  |  |  | |  | |  | Moderate |
| Hafeez et al. (2004) | Y | Y |  |  |  |  |  |  |  |  |  |  |  |  |  |  |  | Y | Y | Y | | Y | | Y | High |
| Hyder et al. (2007) | Y | Y | Y | Y | Y | Y | Y |  |  |  |  |  |  |  |  |  |  |  |  |  | |  | |  | High |
| Ismail et al. (2025) | Y | Y | Y | Y | Y | Y | Y |  |  |  |  |  |  |  |  |  |  |  |  |  | |  | |  | High |
| Khan et al. (2022) | Y | Y |  |  |  |  |  |  |  |  |  |  | Y | Y | Y | U | Y |  |  |  | |  | |  | High |
| Khan et al. (2024) | Y | Y | Y | Y | Y | Y | Y |  |  |  |  |  |  |  |  |  |  |  |  |  | |  | |  | High |
| Lapping et al.  (2002) | Y | Y |  |  |  |  |  |  |  |  |  |  |  |  |  |  |  | Y | Y | Y | | Y | | Y | High |
| Malik et al. (2019) | Y | Y |  |  |  |  |  |  |  |  |  |  | Y | N | Y | N | Y |  |  |  | |  | |  | Moderate |
| Marsh et al. (2002) | Y | Y |  |  |  |  |  |  |  |  |  |  |  |  |  |  |  | Y | Y | Y | | Y | | Y | High |
| Mumtaz (2021) | Y | Y |  |  |  |  |  |  |  |  |  |  |  |  |  |  |  | Y | Y | Y | | U | | U | Moderate |
| Purdin et  al. (2009) | Y | Y |  |  |  |  |  |  |  |  |  |  | Y | Y | Y | Y | Y |  |  |  | |  | |  | High |
| Raheel et  al. (2012) | Y | Y |  |  |  |  |  | Y | Y | Y | Y | Y |  |  |  |  |  |  |  |  | |  | |  | High |
| Shafiq et al. (2025) | Y | Y | Y | Y | Y | Y | Y |  |  |  |  |  |  |  |  |  |  |  |  |  | |  | |  | High |
| Shafiq et al. (2025) | Y | Y |  |  |  |  |  |  |  |  |  |  | Y | Y | Y | Y | Y |  |  |  | |  | |  | High |
| Sumra et al. (2025) |  |  |  |  |  |  |  |  |  |  |  |  | Y | N | Y | N | Y |  |  |  | |  | |  | Moderate |
| Zafar Aga et al. (2025) |  |  |  |  |  |  |  |  |  |  |  |  | Y | Y | Y | Y | Y |  |  |  | |  | |  | High |

#

# **Full-text articles excluded (with reasons for exclusion).**

**Supplementary Table 4**. Post abstract screening cuts.

|  | **Reference** | **Exclusion Criteria** |
| --- | --- | --- |
| 1 | ALGASSEER N, DRESDEN E, KEENEY G, WARREN N. Status of women and infants in complex humanitarian emergencies. Journal of Midwifery & Women’s Health. 2004 Jul;49(4):7–13. | Population not specified or wrong population |
| 2 | Ashraf M, Shahzad S, Sequeria P, Bashir A, Azmat SK. Understanding Challenges Women Face in Flood-Affected Areas to Access Sexual and Reproductive Health Services: A Rapid Assessment from a Disaster-Torn Pakistan. BioMed Research International [Internet]. 2024 Apr 1;2024:e1113634. Available from: https://www.hindawi.com/journals/bmri/2024/1113634/ | Population not specified or wrong population |
| 3 | Bhadra S. Exploring dimensions of sexual issues in disasters and conflicts: Need to bridge the gaps between policy and practice. Sexologies. 2021 Dec; | Population not specified or wrong population |
| 4 | Chang S, Ives B, Oh J. When conflict meets political exclusion: Ethnicity, governance, and child mortality. SSM - Population Health. 2025 Sep;31:101842. | Population not specified or wrong population |
| 5 | Hirani SAA. Barriers to Women’s Menstrual Hygiene Practices during Recurrent Disasters and Displacement: A Qualitative Study. International Journal of Environmental Research and Public Health [Internet]. 2024 Feb 1;21(2):153. Available from: https://www.mdpi.com/1660-4601/21/2/153 | Population not specified or wrong population |
| 6 | Mansukhani R, Haleema Shakur-Still, Chaudhri R, Bello F, Projestine Muganyizi, Kayani A, et al. Maternal anaemia and the risk of postpartum haemorrhage: a cohort analysis of data from the WOMAN-2 trial. The Lancet Global Health. 2023 Aug 1;11(8):e1249–59. | Population not specified or wrong population |
| 7 | Abidi SH, Ali F, Shah F, Abbas F, Ali S. Burden of Communicable Disease among the Native and Repatriating Afghans. Rall GF, editor. PLoS Pathogens. 2012 Oct 25;8(10):e1002926. | Not relevant to Sexual and Reproductive Health / Maternal Welfare |
| 8 | Ahmad J, Morshed Ahmad M, Sadia H, Ahmad A. Using selected global health indicators to assess public health status of population displaced by natural and man-made disasters. International Journal of Disaster Risk Reduction. 2017 Jun;22:228–37. | Not relevant to Sexual and Reproductive Health / Maternal Welfare |
| 9 | Jibeen T. Subjective Well-Being of Afghan Refugees in Pakistan: The Moderating Role of Perceived Control in Married Men. Community Mental Health Journal. 2018 Oct 17;55(1):144–55. | Not relevant to Sexual and Reproductive Health / Maternal Welfare |
| 10 | Kaleem S, Ahmad T, Wahid A, Hamad Haider Khan, Tauqeer Hussain Mallhi, Yaser Mohammed Al-Worafi, et al. Assessment of health-related quality of life among Afghan refugees in Quetta, Pakistan. PloS one. 2024 Feb 1;19(2):e0288834–4. | Not relevant to Sexual and Reproductive Health / Maternal Welfare |
| 11 | Quddus A, Luby SP, Jamal Z, Jafar T. Prevalence of hepatitis B among Afghan refugees living in Balochistan, Pakistan. International Journal of Infectious Diseases. 2006 May;10(3):242–7. | Not relevant to Sexual and Reproductive Health / Maternal Welfare |
| 12 | Rehman AU, Zakar R, Hani U, Fischer F. Sociocultural determinants of health-associated quality of life among Afghan refugees in Pakistan: evidence from a multi-stage cross-sectional study. BMC Public Health. 2025 Mar 5;25(1). | Not relevant to Sexual and Reproductive Health / Maternal Welfare |
| 13 | Saeedullah A, Khan MS, Andrews SC, Iqbal K, Ul-Haq Z, Qadir SA, et al. Nutritional Status of Adolescent Afghan Refugees Living in Peshawar, Pakistan. Nutrients. 2021 Aug 31;13(9):3072. | Not relevant to Sexual and Reproductive Health / Maternal Welfare |
| 14 | Sumra KB, Mumtaz M, Khan NU, Cai HH, Yuan Q. The refugees and health crisis: migration policy management and government response to Afghan migrants. BMC Health Services Research. 2025 Feb 7;25(1). | Not relevant to Sexual and Reproductive Health / Maternal Welfare |
| 15 | Burnham G. Maternal deaths among Afghan refugees. The Lancet. 2002 Feb;359(9307):639–40. | Secondary or Summary Source |
| 16 | Reed RV, Fazel M, Jones L, Panter-Brick C, Stein A. Mental health of displaced and refugee children resettled in low-income and middle-income countries: risk and protective factors. The Lancet. 2012 Jan;379(9812):250–65. | Redundancy (points already covered in included articles) |
| 17 | SCHOOLEY J, MORALES L. Learning From the Community to Improve Maternal–Child Health and Nutrition: The Positive Deviance/Hearth Approach. Journal of Midwifery & Women’s Health. 2007 Jul;52(4):376–83. | Redundancy (points already covered in included articles) |
| 18 | Fatima A, Musharraf S. Cultural bereavement, perceived discrimination, community integration and post-migration stress among Afghan refugees in Pakistan. International Journal of Migration, Health and Social Care. 2026 Jan 1;1–12. | Unable to find full article text |
| 19 | Shah NM, Quddus S. Male migration, women’s decision-making autonomy and reproductive health-care utilization in Pakistan. International Journal of Migration, Health and Social Care. 2025 Feb 14; | Unable to find full article text |
| 20 | Culturally Adapted CMAP Plus LTP for Refugee Mothers with History of Self-harm in Pakistan [Internet]. Clinicaltrials.gov. 2026 [cited 2026 Jan 30]. Available from: https://clinicaltrials.gov/study/NCT05171192 | Unable to find full article text |
